# Tissue engineering strategies for treating intervertebral disc degeneration

**DOI:** 10.3389/fbioe.2025.1582189

**Published:** 2025-07-14

**Authors:** Guangshuai Nie, Weiyang Liu, Fanjia Zeng, Jia Hu, Zhen Wang, Zhonglian Huang, Hongjiang Chen, Jun Hu, Jiankun Xu

**Affiliations:** ^1^ Department of Orthopaedics, The First Affiliated Hospital of Shantou University Medical College, Shantou, Guangdong, China; ^2^ Orthopaedic Medical Research Center, The First Affiliated Hospital, Shantou University Medical College, Shantou, Guangdong, China; ^3^ Musculoskeletal Research Laboratory of Department of Orthopaedics and Traumatology and Innovative Orthopaedic Biomaterial and Drug Translational Research Laboratory, Li Ka Shing Institute of Health, the Chinese University of Hong Kong, Sha Tin, Hong Kong SAR, China; ^4^ Disruptive Innovation Centre for Spatiotemporal Imaging, Li Ka Shing Institute of Health Sciences, Faculty of Medicine, The Chinese University of Hong Kong, Hong Kong, Hong Kong SAR, China

**Keywords:** low back pain, intervertebral disc, intervertebral disc degeneration, stem cells, cell carriers, biological scaffolds, bioactive factors

## Abstract

Low back pain (LBP) is becoming prevalent in clinics, owing primarily to intervertebral disc degeneration (IDD). The mechanism of IDD is complex, and its pathophysiology is primarily characterized by a decrease in intervertebral nucleus pulposus cells and an imbalance in the synthesis and degradation of the extracellular matrix of the intervertebral disc (IVD). Grounded in the concept of regenerative repair, IVD engineering has emerged as a significant research focus in spinal surgery. This review systematically examines the relationship between LBP and IDD, describes the physiological characteristics of healthy IVD and the pathological features of IDD (including inflammatory responses and stress stimulation), and provides an overview of current treatment methods and clinical trials. The review focuses on summarizing and evaluating tissue engineering research, particularly the preclinical and clinical findings on the effects of various seed cells, bio-scaffolds, and bioactive factors on IVD, to explore a more comprehensive therapeutic approach. Lastly, the obstacles and opportunities of tissue engineering repair of the IVD are highlighted.

## 1 Introduction

Low back pain (LBP) is a common clinical symptom that significantly impacts a broad population (Hartvigsen et al., 2018; Hoy et al., 2012). Intervertebral disc degeneration (IDD) is one of the primary causes of LBP (Fontana et al., 2015; Smith et al., 2011; Takatalo et al., 2011). Intervertebral disc (IVD) is a complex structure located between the vertebrae, which consists of three anatomical parts: the nucleus pulposus (NP), the annulus fibrosus (AF), and the cartilaginous endplate (CEP). As the largest avascular organ in the body (Pereira et al., 2013), the main functions of the IVD are to connect adjacent vertebrae, transmit physical pressure, and maintain the movement of the spine. With the onset of IDD, IVD, especially NP, undergoes a progressive dehydration due to proteolytic cleavage of aggrecan together (Karppinen et al., 2011), a substantial reduction of resident cell viability (Buckwalter, 1995; Vergroesen et al., 2015). This ultimately impairs IVD biomechanical properties, subsequently leading to structural alterations and development of discogenic LBP, as well as more severe sequelae, including disc herniation, spinal instability, and stenosis with serious neurological consequences (Roberts et al., 2006).

Current clinical treatments for IDD include conservative approaches and spinal surgery. Conservative treatments, including non-steroidal anti-inflammatory drugs and physiotherapy, can often alleviate the pain. However, the degenerative process of IDD cannot be reversed (Mirza and Deyo, 2007). Spinal surgery to remove the degenerated disc and fuse the adjacent spinal segments may cause degeneration and instability of the neighboring discs (Karppinen et al., 2011). Thus, several efforts are being made to develop innovative approaches to repair or ideally regenerate IVD’s original morpho-functional features.

In recent years, IVD tissue engineering has emerged as a promising therapeutic strategy. The homeostasis of the IVD depends on the interactions of cells, extracellular matrix (ECM), and biomechanical stress (Vergroesen et al., 2015). The pathophysiology of IDD is mainly manifested in the decrease of intervertebral nucleus pulposus cells (NPCs) and the imbalance of synthesis and degradation of ECM. Recovering the ability of the disc to repair the ECM, re-establishing the proteoglycan content, may have a significant therapeutic effect by increasing disc hydration and thereby improving its biomechanics (Vadalà et al., 2015). It is a great therapeutic strategy to increase NPCs by adding exogenous stem cells to degenerative discs (Vadalà et al., 2016; Loibl et al., 2019). In addition, several studies demonstrated that injected mesenchymal stem cells (MSCs) become undetectable in a short period due to premature death after implantation (Vadalà et al., 2012; Acosta et al., 2011), While studies involving MSCs and bio-scaffolds showed better cell survival and differentiation, this could be due to providing exogenous MSCs with the right milieu (Yang et al., 2010; Sakai et al., 2006). To tackle the hostile environment of the degenerated IVD, innovative tissue engineering approaches have been investigated to mimic the original IVD healthy microenvironment by combining cellular therapy, bio-scaffolds, and soluble molecules. Therefore, seed cells, bio-scaffolds, and bioactive factors are pivotal components of disc tissue engineering.

This article aims to comprehensively explore the pathological mechanisms of IDD, current treatment methods, and their clinical trial status, and to focus on the potential and challenges of IVD tissue engineering strategies. By summarizing the latest research findings, this article hopes to provide new ideas and directions for the future treatment of IDD.

## 2 Low back pain and intervertebral disc degeneration

Low back pain (LBP) is one of the most common clinical symptoms, significantly impacting individuals across broad populations (Hartvigsen et al., 2018; Hoy et al., 2012). It is estimated that up to 84% of adults experience LBP at some point in their lives (Walker, 2000). LBP not only severely affects the quality of life of patients but also leads to substantial socioeconomic burdens, becoming one of the leading causes of disability worldwide. Notably, the incidence of LBP is higher in women than in men (Maher et al., 2017).

Intervertebral disc degeneration (IDD) is a leading cause of low back pain (LBP) (Fontana et al., 2015; Smith et al., 2011; Takatalo et al., 2011; Kirnaz et al., 2022). As shown in Figure 2, there are many pathological changes in a degenerated IVD, such as disc height decline and disc herniation. Pathological changes such as herniated intervertebral disc, inflammatory factor infiltration, and nerve ingrowth are closely related to pain (Stefanakis et al., 2012; Khan et al., 2017). Patients often suffer from chronic LBP that may radiate to the buttocks or legs, impairing daily activities. Severe cases can lead to disc herniation, spinal instability, and spinal stenosis.

## 3 The structure and anatomy of IVD

### 3.1 The anatomy and function of IVD

**FIGURE 1:**
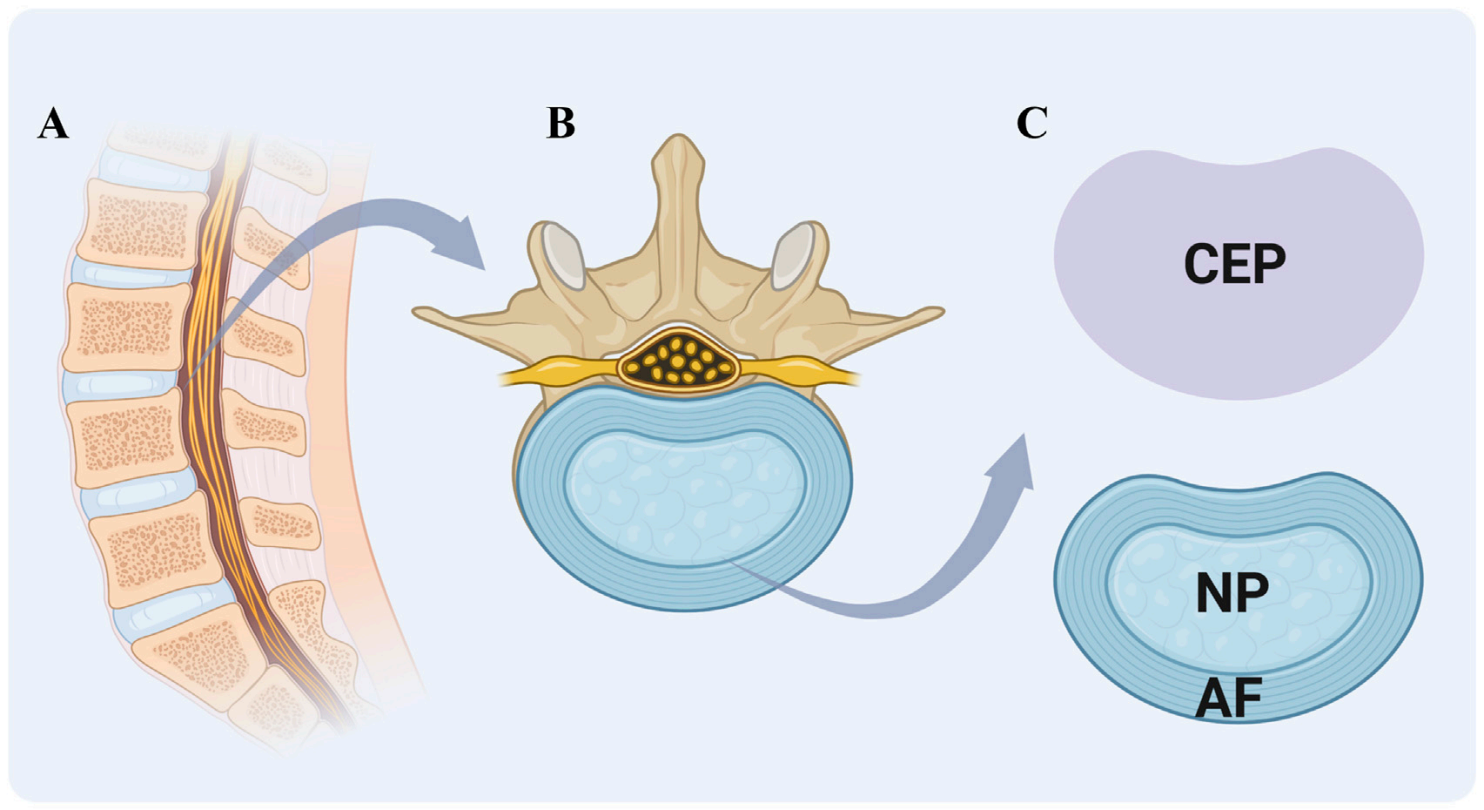
Schematic of Intervertebral Disc (IVD) structure. **(A)** sagittal section of the vertebral column showing the arrangement of vertebrae and intervertebral discs. **(B)** magnified view of a single vertebra-disc unit. **(C)** key components of the IVD include cartilaginous endplate (CEP) (purple region in the upper right), a cartilaginous layer connecting the vertebral body to the disc; gel-like nucleus pulposus (NP), a central region for shock absorption; and fibrous annulus fibrosus (AF), a peripheral ring maintaining structural integrity. Created with BioRender.com.

IVDs articulate the vertebral bodies, constituting approximately one-third of the spinal column’s height and providing mechanical support by facilitating load transfer from body weight and muscle activity. They enable spinal mobility through bending, flexion, and torsion. In the lumbar region of human spine, the size of vertebrae is 7–10 mm in thickness and 4 cm in diameter (Twomey and Taylor, 1987; Roberts et al., 1989). IVDs are complex structures consisting of three specialized tissues: NP, AF, and CEP (Figure 1).

The nucleus pulposus (NP) is a gelatinous tissue characterized by its high hydration and biomechanical properties, predominantly composed of type II collagen and proteoglycans, notably aggrecan. The presence of sulfated glycosaminoglycans (GAGs) within the ECM contributes to its swelling pressure (Urban et al., 1985). Additional proteoglycans in the NP include versican, decorin, biglycan, and fibromodulin, along with significant amounts of elastin, laminin, and fibronectin (Bowles and Setton, 2017). The NP also contains chondrocyte-like cells, termed nucleus pulposus cells (NPCs), which express specific markers such as hypoxia-inducible factor 1α (HIF1α), glypican 3 (GPC3), keratins 8, 18, and 19 (KRT-8/18/19), and paired box 1 (PAX1) (Urban et al., 1985; Bowles and Setton, 2017). Single-cell RNA sequencing has delineated the NPCs into three distinct subtypes: Regulatory Chondrocytes, Homeostatic Chondrocytes, and Effector Chondrocytes. Notably, the Homeostatic Chondrocytes are postulated to play a central role in the biosynthesis of the nucleus pulposus matrix. These cells exhibit high expression of genes associated with ECM synthesis and organization, such as ACAN and COL2A1 (Gan et al., 2021). By synthesizing these matrix components, the NP is enabled to withstand the loads generated by spinal motion and to maintain its highly hydrated state, which is crucial for preserving the physiological function of IVD.

The annulus fibrosus (AF), the exterior component of IVD, encapsulates NP and consists of 15–25 concentric lamellae. These lamellae are predominantly composed of radially and circumferentially oriented type I collagen fibers (Cassidy et al., 1989), which were embedded within an interlamellar matrix rich in non-collagenous proteins, proteoglycans, and fibroblast-like cells (Bruehlmann et al., 2002). Each lamella is formed by strong collagen fibers that incline approximately 30° from one vertebra to the next. The fibers of adjacent lamellae are inclined in the opposite direction at an angle exceeding 60°. This arrangement allows for limited rotation and bending between adjacent vertebrae and enables the intervertebral disc (IVD) to withstand circumferential loads (Mohd Isa et al., 2022).

The thickness of human cartilaginous endplate (CEP) averages approximately 0.6 mm and is structurally akin to hyaline articular cartilage (Roberts et al., 1989). The capillaries of CEP transfer the nutrients in a diffusion manner, from the bone marrow cavities of the vertebral bodies to the inner AF and NP (Grunhagen et al., 2011). Morphologically, the CEP is characterized by elongated cells that are aligned parallel to the IVD and collagen fibers. These cells generate a collagen-rich interterritorial matrix as well as a proteoglycan-rich territorial matrix (Dudli et al., 2016).

### 3.2 Cellular and matrix composition

The IVD exhibits an average cell density of 5.8 × 10^3^ cells/mm^3^, with NP containing 4.0 × 10^3^ cells/mm^3^ and AF having 9.0 × 10^3^ cells/mm^3^. Notably, cellularity within the IVD diminishes progressively with age (Cassidy et al., 1989). In a healthy IVD, cellular anabolism and proliferation are orchestrated by numerous growth factors that are normally present in the disc. These include members of the transforming growth factor β (TGF-β) superfamily, insulin like growth factor 1 (IGF-1), epidermal growth factor (EGF), connective tissue growth factor (CTGF), bone morphogenetic protein-2 (BMP-2), osteogenic protein-1 (OP-1), and growth and differentiation factor-5 and 6 (GDF-5/6) (Kos et al., 2019). Several *in vitro* and *in vivo* studies have showed the capacity of such factors to enhance cell proliferation, proteoglycan, and collagen synthesis while reducing the production of metalloproteinases (MMPs) and proinflammatory cytokines (Kos et al., 2019).

### 3.3 The microenvironment of IVD

The unique anatomical position and function of the IVD confer intrinsic physicochemical properties that create a challenging microenvironment for both resident and exogenously transplanted cells, potentially compromising their viability and functionality (Liang et al., 2012). The IVD is characterized by an avascular, nutrient-poor, hypoxic, acidic, hyperosmolar, and mechanically stressed environment (Wang et al., 2015). These conditions exacerbate with the onset and advancement of IDD (Vo et al., 2016).

## 4 Pathophysiology characteristics of IDD

**FIGURE 2:**
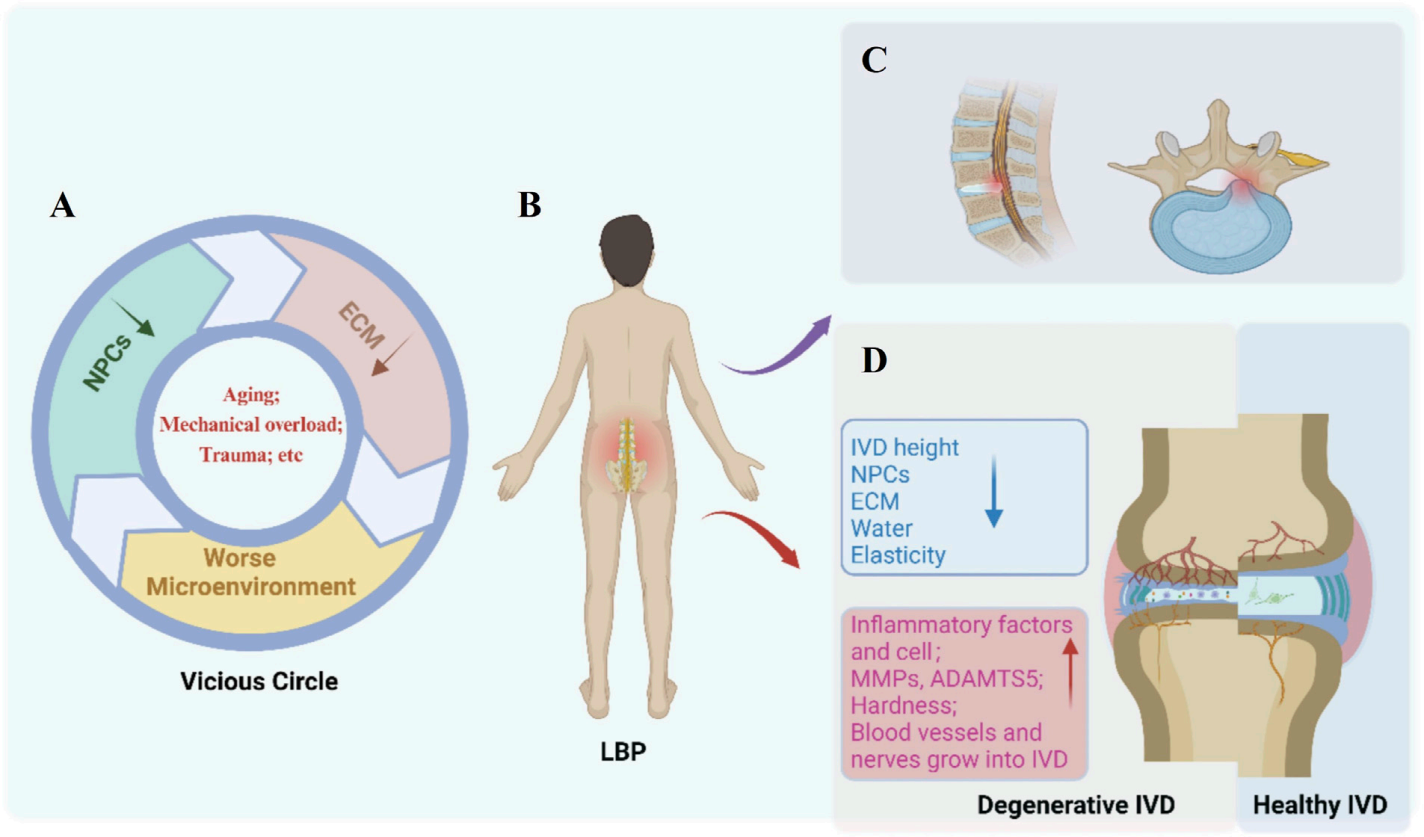
Pathological features of Intervertebral disc degeneration (IDD). **(A)** Etiologies (e.g., aging, mechanical overload, trauma) damage nucleus pulposus cells (NPCs) and extracellular matrix (ECM), initiating a vicious circle of microenvironment deterioration. **(B)** Key molecular and cellular changes in degenerative discs include inflammatory factors, upregulated MMPs/ADAMTS5, tissue hardening, and abnormal vascular/nerve ingrowth. **(C)** Structural degradation manifests as reduced disc height, NPCs loss, ECM disruption, decreased water content, and impaired elasticity. **(D)** Comparative illustration of a degenerative IVD (left) *versus* a healthy IVD (right), highlighting progressive tissue disruption and low back pain (LBP) association. Created with BioRender.com.

IDD has a complicated pathophysiology that is still being unraveled. IDD has a complex etiology that is usually linked to genetic and environmental variables (Feng et al., 2016). Major contributors include aging (Vo et al., 2016), smoking (Nasto et al., 2014), infection (Stirling et al., 2001), mechanical overload (Vadala et al., 2015), trauma (Dudli et al., 2014), obesity (Russo et al., 2017), and diabetes (Russo et al., 2019). The pathophysiology is mainly manifested in the decrease of NPCs and the imbalance of synthesis and degradation of ECM of IVD. The NPCs are responsible for keeping the balance between anabolic and catabolic processes including the synthesis, breakdown, and accumulation of ECM components (Vergroesen et al., 2015). The pathological process of IDD is a vicious circle (Figure 2). Disruption of homeostasis can arise from various etiological factors; for example, trauma may induce apoptosis of NPCs. This cellular demise can subsequently diminish ECM synthesis, which in turn, exacerbates the degradative changes within the IVD microenvironment, perpetuating a cycle that further compromises the viability of NPCs. Calcification of the CEP leads to a reduction in the nutrient supply to the IVD, further exacerbating the progression (Huang et al., 2014).

The structural hallmark of IDD is the progressive dehydration of the NP mainly due to the gradual loss of proteoglycans within the ECM (Vadalà et al., 2015; Vadalà et al., 2018). The principal proteolytic enzymes, MMPs and a disintegrin and metalloproteinase with thrombospondin motifs (ADAMTS) are upregulated (Le Maitre et al., 2007). Some compensations for the proteoglycan loss in the NP with aging occurs by the cells laying down collagens which is more cross-linked, however, this renders the IVD stiffen with less dynamic compliancy under load (Duance et al., 1998). Although the total collagen content of the disc changes little with disc degeneration, a shift in the proportions of collagen types and in their distributions within the matrix changes, that is, decrease of type II collagen and increase of type I collagen. Furthermore, the fibrillar type I and II collagens, become more denatured in degenerated IVDs (Antoniou et al., 1996). These matrix changes may render the degenerated IVD as a more brittle stiffer tissue with less dynamic compliancy properties under load and more prone to the development of fissures in the IVD, further compromising its normal biomechanical functions (Vergroesen et al., 2015). The pathophysiological changes of IDD mainly include inflammation and biological stress.

### 4.1 Inflammatory reaction

IDD is closely related to inflammatory reaction. Inflammation of IVD may be characterized by elevated expression of proinflammatory molecules such as interleukins (IL-1, IL-2, IL-4, IL-6, IL-8, IL-10, and IL-17), TNF-α, IFN-γ, chemokines and prostaglandin E2 (PGE2), leading to the development of IDD and LBP (Molinos et al., 2015; Lyu et al., 2021). TNF-α, IL-1, IL-6 and IL-17 are increased in either the IVD or peripheral serum in patients (Ding et al., 2017; Gruber et al., 2013; Risbud and Shapiro, 2014; Deng et al., 2016; Suzuki et al., 2017). Such inflammatory molecules induce cell apoptosis, senescence and autophagy and upregulate the synthesis of MMPs (−1, −3, −7, −9 and −13) and ADAMTS (−1, −4, −5, −9 and −15) through NPCs, thus leading to ECMs catabolism (Risbud and Shapiro, 2014). TNF-α and IL-1β are key inflammatory mediators of IDD, positively correlating with the severity and promoting ECMs catabolism by up-regulating proteolytic enzymes, whilst inhibiting the expression and synthesis of proteoglycan (Le Maitre et al., 2005; Doita et al., 2001; Jimbo et al., 2005).

### 4.2 Biomechanics: a reduction in intradiscal pressure

In a healthy IVD, the negative charge of the proteoglycan produces osmotic potential, which is transformed by the attraction of water into a biomechanical hydrostatic pressure. Intradiscal pressure stretches the AF and supports the endplate during axial compression and is hence a primary determinant of disc height and stiffness (Vergroesen et al., 2014; Brinckmann and Grootenboer, 1991). In a degenerative IVD, the increased fragmentation of aggrecan reduces the negative charge, thereby reducing pressure within the disc and the ability to retain water under pressure (Vergroesen et al., 2014; Sato et al., 1999; Masuoka et al., 2007; Iatridis et al., 2013). A degenerated IVD can be analogous to a deflated tire, characterized by diminished disc height and compromised axial compressibility, along with augmented and variably unstable radial bulges (Vergroesen et al., 2014; Brinckmann and Grootenboer, 1991; Masuoka et al., 2007).

## 5 Clinical treatment and limitations of LBP caused by IDD

Intervertebral disc degeneration (IDD) is one of the main causes of chronic low back pain (LBP). Current treatment strategies primarily include non-surgical and surgical approaches. However, existing methods mostly focus on alleviating symptoms and slowing disease progression, rather than fundamentally repairing the IVD structure or restoring its function.

### 5.1 Non-surgical treatments

Non-surgical treatments are the first-line approach for low back pain associated with IDD, encompassing bed rest, physical therapy, acupuncture, tuina (massage), and pharmacological interventions (Mirza and Deyo, 2007). While these methods can provide symptomatic relief, they have limited capacity to repair the underlying disc degeneration and are often associated with side effects. For instance, non-steroidal anti-inflammatory drugs (NSAIDs) are effective in reducing pain and inflammation but may cause gastrointestinal bleeding, renal damage, and cardiovascular issues with long-term use (Arfeen et al., 2024). Traditional Chinese medicine treatments, such as acupuncture and tuina, have some efficacy in symptom management but carry risks of misuse.

### 5.2 Surgical treatments

Surgery is the main option for patients with severe symptoms or those who fail non-surgical treatments, including lumbar discectomy and spinal fusion. Lumbar discectomy (whether open or minimally invasive) can alleviate some symptoms but poses potential risks of residual nucleus pulposus, nerve injury, and postoperative instability (Karppinen et al., 2011). Moreover, 5%–24% of patients undergo a second surgery due to recurrent disc herniation (Peng et al., 2024). Spinal fusion surgery, while effective in relieving pain, is highly invasive, limits spinal mobility, and may accelerate degeneration in adjacent segments, a phenomenon known as adjacent segment disease (ASD). The incidence of ASD typically ranges from 2% to 36%, depending on the specific spinal region involved (such as the lumbar or cervical spine) and the duration of follow-up (Huang et al., 2025).

## 6 Current trends in clinical trials on IDD

With the accelerating global aging process, clinical research on intervertebral disc degeneration (IDD) has shown significant growth. Our research team conducted a comprehensive search of the *Clinical* Trials.gov database using keywords including “Intervertebral Disc Degeneration” and related terms, identifying a total of 576 registered clinical studies. From these, we selected the 100 most recent clinical trials for in-depth analysis, with 95 studies ultimately meeting the inclusion criteria. These findings clearly demonstrate the increasing scientific investment and clinical demand in this field.

The analysis of clinical trials revealed a distinct distribution across five major therapeutic approaches for IDD. Pharmacological therapies accounted for 15% (14 studies) of interventions, primarily featuring collagenase injections (NCT06995066) and stem cell-based treatments (NCT06589271). Surgical interventions represented the largest proportion at 30% (28 studies), with a strong emphasis on minimally invasive techniques such as endoscopic discectomy (NCT06615518) and disc replacement procedures (NCT06989632). Rehabilitation therapies comprised 25% (24 studies) of the total, focusing on non-pharmacological interventions including core stabilization exercises (NCT06969456). Observational studies made up 20% (19 studies), predominantly examining postoperative outcomes and imaging-based assessments (NCT06778447). Comprehensive treatment strategies, which integrated pharmacological, surgical, and rehabilitation approaches, constituted the remaining 10% (10 studies) of the analyzed trials. This distribution highlights the current therapeutic landscape, where surgical and rehabilitation approaches dominate, while combination therapies and pharmacological innovations continue to develop.

Recent trends in intervertebral disc degeneration research reveal three key developments: minimally invasive techniques, particularly robotic-assisted and endoscopic surgeries, have become the predominant surgical approach; non-pharmacological interventions including targeted exercise regimens and laser therapy are attracting growing research attention; and innovative biological therapies, especially stem cell applications and advanced biomaterial implants, are emerging as promising novel treatment directions. These trends collectively demonstrate a shift toward less invasive procedures, greater emphasis on conservative management, and increasing exploration of regenerative medicine solutions in the field of disc degeneration therapeutics.

## 7 Why tissue engineering might be a better option

The treatment of intervertebral disc degenerative diseases has long relied primarily on conservative therapies and surgical interventions. However, these traditional methods, despite providing some symptomatic relief, have significant limitations (Arfeen et al., 2024; Huang et al., 2025). They are unable to address the root cause of disc degeneration or restore the biological integrity and mechanical properties of the intervertebral disc.

In contrast, tissue engineering, through its synergistic “cell-scaffold-bioactive factor” approach, has demonstrated revolutionary advantages. For instance, the combination of BMSCs and alginate scaffolds has been proven in animal models to effectively activate endogenous NPCs and promote the differentiation of BMSCs into NPCs, while stimulating the production of growth factors and ECM (Ukeba et al., 2020). This process holds the potential for true regeneration of the intervertebral disc.

Tissue engineering not only promotes tissue regeneration through the combination of cells and scaffolds but also optimizes therapeutic outcomes through the sustained release of bioactive factors. This integrated approach not only overcomes the limitations of traditional treatments but also provides a more comprehensive and durable solution for intervertebral disc degenerative diseases (Russo et al., 2021; Steffen et al., 2019). Therefore, tissue engineering is undoubtedly a key focus for future research and has the potential to fundamentally change the treatment paradigm for intervertebral disc degeneration.

## 8 Any clinical trials in tissue engineering

Our research team conducted a systematic search of the ClinicalTrials.gov database using professional terminology combinations including “intervertebral disc degeneration,” “tissue engineering,” “regenerative medicine,” and related terms identifying a total of 71 relevant registered clinical trials. Through the establishment of strict inclusion criteria, we ultimately determined that 18 studies met the core definition of intervertebral disc tissue engineering therapy. While there may be some unregistered or unretrieved related studies, the existing data sufficiently reflects the development trends and main research directions in this field.

Current clinical research is primarily focused on two major areas: stem cell therapy and bioactive delivery systems. Stem cell therapy emphasizes the application of autologous bone marrow mesenchymal stem cells (e.g., NCT05066334) and umbilical cord mesenchymal stem cells (e.g., NCT06589271). The cell-scaffold composite technology (e.g., NCT03002207) is also widely used to enhance the survival rate and functional maintenance of transplanted cells. In the field of bioactive delivery systems, research mainly explores novel delivery carriers, such as PRP-exosome complexes (e.g., NCT04849429) and BMP-2 hydrogels (e.g., NCT01106417).

## 9 The tissue engineering strategies for tackling IDD

**FIGURE 3:**
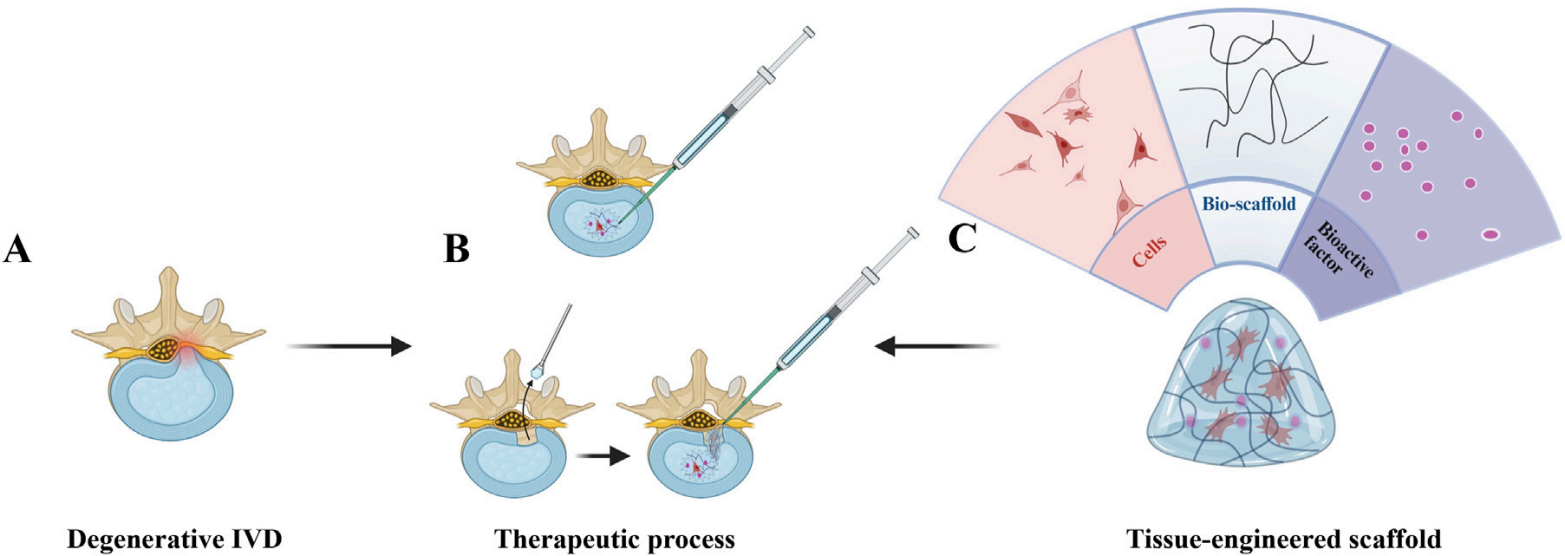
Regenerative treatment strategy for intervertebral disc degeneration (IDD). **(A)** Degenerated disc (lower-left) exhibiting structural disruption (e.g., height loss, annular tears) and inflammatory microenvironment. **(B)** Two delivery approaches: direct intradiscal injection (upper-left syringe) and post-annulotomy injection (horizontal-right syringe), administering a regenerative mixture (cells + bio-scaffold + bioactive factors). **(C)** Functional analysis (right-side fan diagram): Cells drive tissue repair, bio-scaffold provides Three-dimensional growth support, and bioactive factors suppress inflammation and stimulate ECM synthesis, collectively restoring disc integrity (partial/complete). Created with BioRender.com.

Regenerative strategies on the IVD are based on three main components, seeding cells, bio-scaffold and bioactive factors, which can be utilized separately or together to promote repair (Figure 3). The main principle is that seed cells, such as marrow mesenchymal stem cells (MSCs), are implanted into the disc to replenish the IVD cells. Although the exact mode of action of MSCs is unknown, they can either repair IVD directly or stimulate the growth of IVD cells by secreting growth factors. However, due to the inhospitable internal environment of IVD, multiple investigations have shown that the injected MSCs quickly become undetectable (Vadalà et al., 2012; Acosta et al., 2011). Bio-scaffolds or microcarriers can provide direct mechanical support and provide an environment that allows cells to survive, proliferate and differentiate. Biomolecules (genes, growth factors, cytokines) stimulate the resident IVD cells and maintain the active state of administered seeding cells, facilitating the repair process (Chu et al., 2018). Depending on whether the AF has been destroyed, the type of injection and the choice of the scaffold may vary. For example, some studies have focused on disc regeneration after NP resection, so they can choose a light-cured hydrogel as a bio-scaffold.

### 9.1 Stem cells for the IVD repair

Various types of MSCs can be integrated with regenerative strategies for IDD. Table 1 summarizes the tissue origins of the applied cells, including MSCs isolated from the AF (Zhu et al., 2016), NP (Ukeba et al., 2020), Whartons jelly (Choi et al., 2020), umbilical cord (Beeravolu et al., 2018) and cartilaginous tissue (Beeravolu et al., 2018) as well as the more common adipose (Xiao et al., 2021) or bone marrow mesenchymal (Lykov et al., 2020). Bone MSCs (BMSCs), which were discovered in the 1960s, have been studied extensively and used more than any other stem cell types. A growing number of preclinical studies have confirmed the safety, feasibility and efficacy of intradiscal cell therapy based on BMSCs, thus laying a foundation for clinical application. Adipose-derived MSCs (ADSCs) have clinical advantages, such as their minimally invasive and safe accessibility, high frequency in adipose tissue and high proliferation rate (Pereira et al., 2013; Karppinen et al., 2011). ADSCs are more readily available than BMSCs, given that adipose tissue is a more abundant source of stem cells than bone marrow and liposuction is more acceptable for bone marrow aspiration. So even in the future, the ability of ADSCs to differentiate into chondrocytes and tolerate acid and hyperosmotic microenvironments is lower than that of BMSCs, high ADSCs proliferation and low donor site morbidity may be beneficial to their application (Liang et al., 2012).

**TABLE 1:** Different seeding cells in IVD regeneration strategies.

Cell species	Cell source	Engineered MSCs	Model	Advantages	Disadvantages	Ref.
ADSCs	Human		Rat IDD model	Plentiful source; strong proliferation; differentiation and immunomodulation capabilities (Strioga et al., 2012)		Yu et al. (2021)
	Human	HADSCs overexpressing Sod2 and Cat	Mouse IDD model			Xiao et al. (2021)
	Rat	ADSC-TEC	Rat IDD model			Ishiguro et al. (2019)
BMSCs	Rat		Rat IDD model	Plentiful source, superior osteogenic and chondrogenic differentiation potential (Strioga et al., 2012)	Aspiration of bone marrow is a painful procedure	Lykov et al. (2020)
	Rabbit		Rabbit IDD model			Ukeba et al. (2020)
	Rabbit	Co-culture of BMSC with MECs	Rabbit IDD model			Zhang et al. (2018)
	Rabbit	BMSCs overexpressing BMP7	Rabbit IDD model			Xu et al. (2016)
	Rabbit	BMSCs overexpressing BMP2	Rabbit IDD model			Hou et al. (2016)
	Rabbit	BMSCs overexpressing CXCR4	Rabbit IDD model			Wei et al. (2016)
	Human	Co-culture of BMSCs with NPCs	Patients with IDD			Mochida et al. (2015)
WJMSCs	Human		Rat IDD model	Hypoimmunogenicity and immunomodulatory potential (Batsali et al., 2013)	Donor shortage	Choi et al. (2020)
	Human		Rabbit IDD model			Ahn et al. (2015)
DFs	Human and Rabbit		Rabbit IDD model	Easy access		Shi et al. (2019)
UCMSCs	Human		Rabbit IDD model	Strong proliferation and differentiation ability	Donor shortage	Beeravolu et al. (2018)
CPCs	Human		Rabbit IDD model	Strong chondrogenic differentiation potential (Jayasuriya and Chen, 2015)		Beeravolu et al. (2018)
NPCs	Rabbit		Rabbit IDD model	Ideal cell	Donor shortage	Ukeba et al. (2020)
AFSCs	Rabbit		Synthetic AF tissue	Ideal cell	Donor shortage	Zhu et al. (2016)

AF, annulus fibrosus; AFSCs, Annulus fibrosus-derived stem cells; CPCs, Chondroprogenitor cells; DFs, Dermal fibro-blasts; IDD, intervertebral disc degeneration; IVD, intervertebral disc; MECs, Microencapsulated Chondrocytes; MSCs, mesenchymal stem cells; ADSCs, Adipose-derived MSCs; **NPCs**, Nucleus Pulposus Cells; **NPCs**, Nucleus Pulposus; UCMSCs, Umbilical cord mesenchymal stem cells; WJ-MSCs, Whartons jelly mesenchymal stem cells. CXCR4: C-X-C motif chemokine receptor four.

NP-MSCs tend to exhibit higher chondrogenic differentiation than BMSCs and ADSCs, considering that residual cell phenotypes are more readily available from MSCs isolated from specific tissues (Fang et al., 2009). NPCs are probably the most effective cells for IDD and studies have shown that NPCs are very effective in promoting IVD regeneration and ECM synthesis (Ukeba et al., 2020). WJ-MSCs, which have low immunogenicity and immunomodulatory potential, have gained great interest in clinical application (Choi et al., 2020; Ahn et al., 2015). So WJ-MSCs have been used in disc degeneration, either alone or in combination with hydrogels and has been shown to be effective in disc repair (Choi et al., 2020; Ahn et al., 2015). Other sources of cells, such as UCMSCs and CPCs, have also been shown to play an active role in disc regeneration in degenerative disc disease. Considering the cell-harvesting aspect, it is difficult to apply other cell types on a large scale except BMSCs and ADSCs. Therefore, BMSCs and ADSCs are the most useful tissue regeneration options.

To further enhance the effectiveness of MSCs, many engineered cells have been developed that overexpress key proteins that promote proliferation and matrix production in IVD cells, including Bone morphogenetic protein 7 (BMP7) (Xu et al., 2016), Bone morphogenetic protein 2 (BMP2) (Hou et al., 2016), (C-X-C chemokine receptor type 4 (CXCR4) (Wei et al., 2016), superoxide dismutase 2 (Sod2) and catalase (Cat) (Xiao et al., 2021). In addition, there are other research attempts, such as hypoxic or pentosan polysulfate pretreatment of mesenchymal cells (Daly et al., 2018; Ejtehadifar et al., 2015), co-culture of BMSCS and MECs (Zhang et al., 2018) and use of ADSCs to construct scaffold-free tissue engineering (ADSC-TEC) (Ishiguro et al., 2019).

Clinical applications have increased in recent years as more preclinical studies have shown the safety, feasibility, and efficacy of MSC-based cell therapy. Henriksson et al. (Henriksson et al., 2019) tested the feasibility study of transplanting MSCs into degenerative discs in four patients. Results demonstrated that MSCs labelled with iron sucrose remained detectable at 8 months post transplantation. Centeno et al. (Centeno et al., 2017) studied 33 patients with LBP and radiculopathy and injected autologous BMSCs. Pain and reported function (FRI) were significantly reduced at 3, 36, 48, 60, 72 months after injection. Noriega, D. C et al. (Noriega et al., 2017) conducted a study involving the intra-discal injection of allogeneic BMSCs in patients with IDD unresponsive to conservative management. The outcomes demonstrated a rapid and significant improvement in functional indices for the treatment group. It indicates that allogeneic MSCs may offer a therapeutically equivalent and logistically more convenient alternative to autologous MSCs for the treatment of IDD.

Overall, cell therapy has been shown to be safe in all previous reports and no major adverse events have been observed (Meisel et al., 2019). In addition, as reported in some clinical studies, cell therapy is even also effective in reducing pain and promoting regeneration (Centeno et al., 2017; Noriega et al., 2017; Pettine et al., 2015).

### 9.2 Biological scaffolds or microcarriers for IVD repair

Biological scaffolds or microcarriers, predominantly in the form of hydrogels or microspheres, encompass natural materials, synthetic materials and composite materials. Their functions include the delivery of cells and the provision of an environment conducive to cell survival, proliferation and differentiation. They also serve to deliver active factors and pharmaceuticals, with a sustained release effect. Ultimately, the scaffold itself can act as a supplement to ECM of the IVD, offering mechanical support. Ideal biomaterials for scaffold should meet the following requirements: a). limiting MSCs leakage; b). supporting the survival of exogenous cells and remaining NPCs in the intervertebral disc; c). promoting the formation and deposition of ECM in the IVD; d). enhancing spinal stability; e). lowering inflammation; f). sustained release effect.

#### 9.2.1 Cell delivery vehicles

In the application of MSCs transplantation, the adverse microenvironment within IVD may impede the growth and differentiation of the MSCs, potentially compromising the therapeutic efficacy. The leakage of MSCs at the implanted site also hinders its application. Many research teams have studied and prepared various biomaterials to address these issues and many cell delivery vehicles have been developed for the administration of MSCs and other therapeutic cells into the degenerated IVD (Table 2).

**TABLE 2:** Cell carriers used in IVD repair strategies.

Materials Category		Composition	Cell species	Features	Ref.
Natural materials	UPAL gel	Alginate	MSCs	Abundant raw materials; high biocompatibility; Limited stability and mechanical properties	Ukeba et al. (2020), Wang et al. (2014), Bertolo et al. (2015)
	HA	HA	ADSCs, WJMSCs	Abundant raw materials; high biocompatibility; Limited stability and mechanical properties	Ahn et al. (2015), Kumar et al. (2017)
	Gelatin sponge	Gelatin	MPC, BMSCs	Enough safety; abundant raw materials; Limited stability and mechanical properties	Daly et al. (2018), Yuan et al. (2022)
	HDC gel	Collagen	BMSCs	Enough safety; abundant raw materials; good efficacy in annular repair	Hussain et al. (2019)
	Decellularized NP scaffold	ECM (Collagen, GAGs)	HBMSCs	Good cytocompatibility; similar NP ECM biological components and microstructure Donor shortage	Xu et al. (2019a), Fernandez et al. (2016)
	Porous silk fibroin scaffold	Silk fibroin	Rabbit NPCs	Good biocompatibility; proper mechanical properties; low immunogenicity; controllable degradation rate	Zeng et al. (2014)
	Small intestinal mucosa		Human degenerative AFCs and NPCs	Porous structure; the entrapped growth factors; biocompatible and biodegradable Donor shortage	Le Visage et al. (2006)
Synthetic/Composite materials	PPCC hydrogel	PLLA, PLGA, CH, CS	BMSCs	Nanofibrous Structure; Laser Micromachining; high biocompatibility	Nakielski et al. (2023)
	GFA	GelMA, Foxy5-MA, AOP-MA	BMSCs	Anti-inflammatory; antioxidant	Chen et al. (2025)
	TBA@Gel&Chs microspheres	TBA, Gelatin, Chondroitin sulfate	NPCs	Antioxidant Unpredictable degradation *in vivo*	Yang et al. (2025a)
	SA/PNIPAAm hydrogel	SA, PNIPAAm, SC	NPCs	Ion-Controlled Release: Ca^2+^ and Mg^2+^	Jiang et al. (2023)
	HA and PRP Hydrogel	HA, PRP	HBMSCs	Rich in growth factors; enough safety; abundant raw materials; high biocompatibility	Russo et al. (2021)
	HAMC hydrogel	HA, MC	WJ-MSCs	Biodegradable, water soluble; injectable	Choi et al. (2020)
	Self-assembling peptide hydrogel	GO, β-sheet forming Self-assembling peptide	Bovine NPCs	Biocompatible and non-immunogenic nature; injectable; mechanical properties similar to the NP	Ligorio et al. (2019)
	Dextran/Gelatin Hydrogel	Dextran, Gelatin	Mouse BMSCs	Biocompatible; injectable; sustained release TGF-β3	Gan et al. (2016)
	Genipin cross-linked type II collagen/CS composite hydrogel	Genipin, Collagen, CS	ADSCs	Injectable; biocompatible; low cytotoxicity	Zhou et al. (2018)
	the RADKPS functional peptide	RADA16-I, BMP-7	BMSCs	No cytotoxicity; injectable; biocompatible; conjugating a bioactive motif derived from BMP-7	Wu et al. (2016)
	Ultraviolet photocurable hydrogel	PHEMA, APMA, PAA	HBMSCs	Photocurable; injectable	Kumar et al. (2016)
	SAPHs	Phenylalanine, glutamic acid, lysine	Bovine NPCs	The similar mechanical properties as the native NP; injectable; biocompatible	Wan et al. (2016)
	Thermally triggered hydrogel	NIPAM, DMAc, Water	HBMSCs	Injectable; body temperature triggers gelation	Thorpe et al. (2016)
	CP with CS hydrogels	CP, CS	Rat ADSCs	Biocompatible; enough mechanical strength	Nair et al. (2015)

APMA, aminopropylme thacrylamide; AF, annulus fibrosus; ADSCs, Adipose-Derived Stem Cells; BMP-7, Bone morphogenetic protein-7; BMSCs, Bone Marrow Mesenchymal Stem Cells; CH, chitosan; CS, chondroitin sulfate; CP, Chitosan-poly (hydroxybutyrate-co-valerate); DMAc, Dimethylacrylamide; GO, graphene oxide; GFA, Gelatin Methacryloyl Matrix Grafted with Wnt5a-mimetic Peptide Foxy5 and Antioxidative Peptide Hydrogel Microspheres; HDC, high density collagen; HBMSCs, Human BMSCs, HA, hyaluronic acid; HAMC, hyaluronan-methylcellulose; MC, methylcellulose; MPC, mesenchymal progenitor cells; NPCs, Nucleus Pulposus Cells; NP, nucleus pulposus; NIPAM, N-isopropylacrylamide; PRP, Platelet-rich plasma; PHEMA, Poly-hydroxyethyl methacrylate; PAA, polyacrylic acid; PNIPAAm, poly (N-isopropylacrylamide); PLLA, Poly (L-Lactide); PLGA, Poly (L-lactide-co-glycolide); SA, sodium alginate; SAPHs, Self-assembling peptide hydrogels; SC, silicate ceramics; TGF-β3, Transforming Growth Factor Beta 3; TBA@Gel&Chs microspheres, 2,3,4- trihydroxy benzaldehyde (TBA), Gelatin (Gel) and chondroitin sulfate (Chs); UPAL, Ultra-purified alginate.

The sources of materials for cell vehicles can be classified into natural, synthetic and composite types. Natural materials, including silk fibroin (Zeng et al., 2014), fibrin (Buser et al., 2011), collagen (Huang et al., 2010), ECM (Borem et al., 2017), Gelatin (Daly et al., 2018; Yuan et al., 2022), alginate (Chou and Nicoll, 2009), hyaluronic acid (HA) (Huang et al., 2011), and chitosan (Roughley et al., 2006) have demonstrated with biocompatibility and minimal cytotoxicity. However, natural hydrogels often face issues with inadequate stability and insufficient mechanical strength, which can limit their effectiveness in certain applications (Hu et al., 2025). The synthetic materials, including polylactic acid, polyglycolic acid, polyurethane, poloxamer, polycaprolactone, polyethylene glycol (Bowles and Setton, 2017; Mizuno et al., 2004; Kondiah et al., 2016), display excellent controllability, easy to design, and good mechanical qualities (Wismer et al., 2014; Helen et al., 2007). However, synthetic hydrogels may lack biocompatibility and can be toxic when certain polymers react, significantly limiting their use in medical treatments (Hu et al., 2025). Composite materials, which combine natural and synthetic materials to retain the benefits of both, such as good mechanical qualities and biocompatibility, are the IVD regeneration and replacement’s future research directions.

##### 9.2.1.1 Cell delivery vehicles from natural materials

Due to good biocompatibility and low cytotoxicity, many scaffolds and hydrogels assembled from natural polymers are widely used in the regeneration of degenerated IVD. HA hydrogels are extensively investigated for their myriad of beneficial properties, which include an abundance of raw materials, exceptional biocompatibility and potent anti-inflammatory effects (Day and de la Motte, 2005). The use of HA hydrogels in biomedical applications is particularly favored due to their innate presence in the human body, which contributes to their high degree of compatibility with biological tissues. Furthermore, the hydrophilic nature of HA confers to the hydrogel its unique ability to retain substantial amounts of water, providing a hydrated microenvironment that is conducive to cell growth and tissue repair.

Hyaluronic acid (HA), a glycosaminoglycan (GAG), is inherently a component of the ECM of the NP. Consequently, HA can serve as a supplement to the NP, providing mechanical support. The combined use of MSCs and HA scaffolds more effectively supports IVD regeneration than the use of MSCs or HA scaffolds alone in a rabbit model of IDD (Ahn et al., 2015). Kumar et al. (Kumar et al., 2017) enrolled 10 eligible chronic LBP patients who underwent a single intradiscal injection of combined HA derivative and ADMSCs and performed a 12-month follow-up. No surgical or stem-cell related adverse events or severe adverse events were observed. Significant improvements in the VAS pain score and ODI score were demonstrated in six of 10 patients. However, the limited stability and mechanical properties of HA restrict its direct application, leading researchers to commonly employ HA-based modified hydrogels for treating intervertebral disc degeneration. (Guo et al., 2022).

Alginate, a polysaccharide derived from natural sources, is commonly used to create hydrogels crosslinked by divalent calcium ions. Similar to HA hydrogels, alginate hydrogels are extensively studied for their potential in IVD degeneration repair and cell delivery systems. In a study by Ukeba et al. (Ukeba et al., 2020), the implantation of BMSCs with UPAL gel into degenerative IVD rabbit models showed BMSC viability for 12 weeks and improved IVD degeneration, as confirmed by MRI. The findings suggest that BMSCs in conjunction with UPAL gel enhance IVD regeneration by stimulating endogenous NPC activity and ECM production. It shows potential in IDD repair and cell delivery systems but has a low compressive strength. Additionally, concerns regarding its long-term safety and potential toxicity remain due to inadequate understanding of its long-term effects. (Arfeen et al., 2024).

Chitosan, a product of the N-deacetylation of chitin, possesses various advantages inherent to natural substances, such as biocompatibility, biodegradability, as well as non-toxicity and antimicrobial properties. These characteristics have led to its extensive applications in the fields of medical devices and as a scaffold material in tissue engineering (Bhattarai et al., 2010). However, traditional chitosan hydrogels have weak mechanical properties, limiting their application in biomedical fields (Wang et al., 2025).

Because of complex components and 3D structure of NP, many studies focus on acellular NP scaffolds. Acellular protocols usually remove the cellular components of NP, retaining most of the biological components and regular microstructures. Xu et al. (Xu J. et al., 2019) developed an optimized decellularized NP scaffold that could induce the differentiation of MSCs towards NPCs and serves as a potential treatment for degenerated IVDs. The challenge of effectively removing cellular debris to prevent immune reactions while maintaining the biological integrity of the ECM requires further investigation. Tissue origin and storage conditions prior to decellularization significantly impact the quality of decellularized extracellular matrix (dECM, leading to variability between batches, even among the same tissue types (Soltanmohammadi et al., 2025).

Vadala et al. (Vadalà et al., 2012) injected MSCs into IVDs in a rabbit model of degeneration and observed large osteophytes at the injection sites, suggesting that MSCs may unexpectedly cause bone hyperplasia. For AF defects, MSCs are not confined to the injection site, which are transferred via normal saline or PBS carriers. Hussain et al. (Hussain et al., 2019) developed an riboflavin crosslinked, high-density collagen (HDC) gel to deliver MSCs into AF defects in a safe and effective manner for annular repair in a sheep lumbar IVD injury model and improved outcomes in terms of DHI, Pfirrmann grade and T2-RT. Daly et al. (Daly et al., 2018) used gelatin sponge as cell carrier and used fibrin sealant to block the outer region, which achieved comparable effect.

**FIGURE 4:**
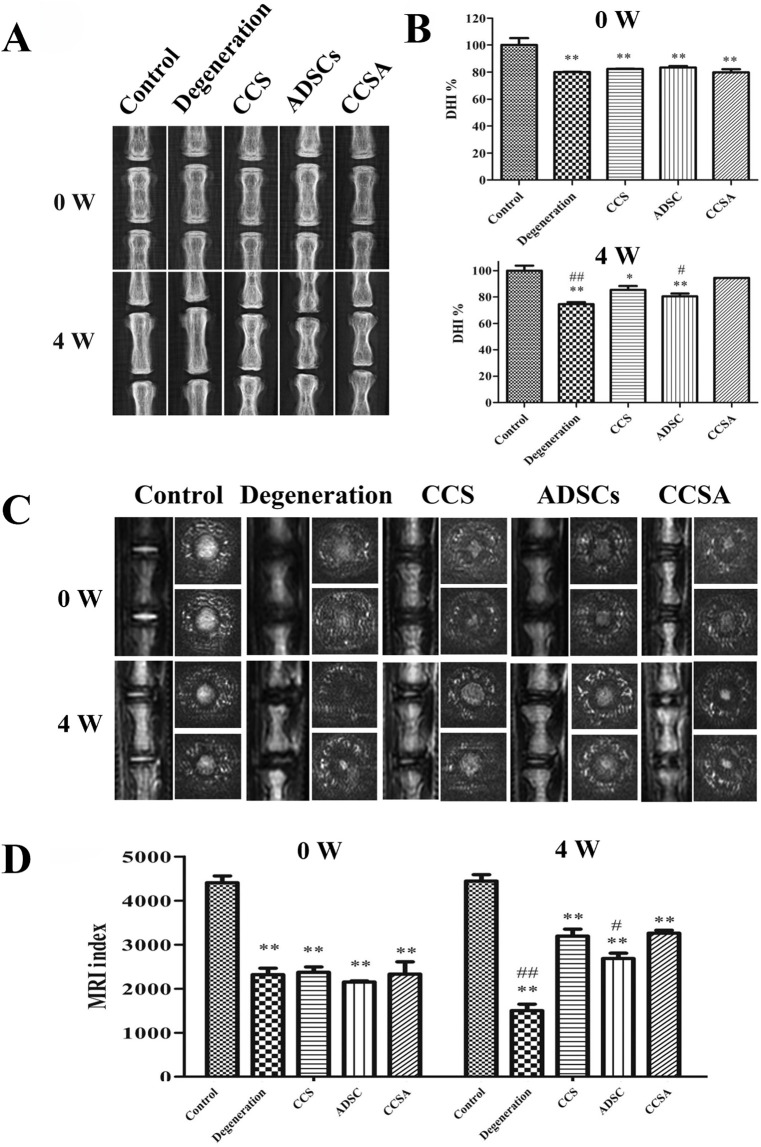
Therapeutic effects of type II collagen/chondroitin sulfate (CS) composite hydrogel-like ADSC (CCSA) delivery system on intervertebral disc degeneration in a rat tail model. **(A)** Representative radiographs showing coccygeal vertebral discs at 0 and 4 weeks post-injection in five experimental groups: Control, Degeneration, CCS (collagen/chondroitin sulfate hydrogel), ADSCs (adipose-derived stem cells), and CCSA (composite hydrogel with ADSCs). **(B)** Quantitative analysis of disc height index (DHI%) demonstrating significant restoration of disc height in the CCSA group compared to degeneration controls. **(C)** Corresponding T2-weighted Magnetic Resonance Imaging (MRI)scans revealing morphological changes in nucleus pulposus hydration status. **(D)** MRI index quantification confirming partial restoration of disc hydration and structure in the CCSA-treated group. Reproduced with permission (Zhou et al., 2018). Copyright 2018, Elsevier.

To transplant ADSCs into the NP and maintain cell function, Zhou et al. (Zhou et al., 2018) developed a type II collagen/chondroitin sulfate (CS) composite hydrogel-like ADSC (CCSA) delivery system with genipin as the cross-linking agent. After the injection of the CCSA system, the disc height, MRI index, water content, ECM synthesis and structure of the degenerated NP were partly restored (Figure 4).

Hydrogels composed of a single natural polymer possess distinct advantages in terms of tissue compatibility and cytotoxicity safety. However, they often exhibit performance limitations, such as insufficient mechanical strength in pure fibrin hydrogels (Liu et al., 2023). Consequently, in recent studies, natural polymers are frequently utilized as components in composite hydrogels to complement the properties of other hydrogel constituents.

##### 9.2.1.2 Cell delivery vehicles from synthetic or composite materials

Due to the limited functionality and unsatisfactory mechanical properties of natural materials, current mainstream research focuses on the use of synthetic or composite materials as carriers for cellular delivery. For instance, Chitosan (CH) gels exhibit inferior mechanical properties and extended gelation times, which limits their standalone use. Consequently, they can be integrated into composite hydrogels with materials such as poly (L-lactide-co-glycolide) (PLGA), poly (L-lactide) (PLA) and chondroitin sulfate (CS) to enhance the resulting hydrogel system’s performance.

**FIGURE 5:**
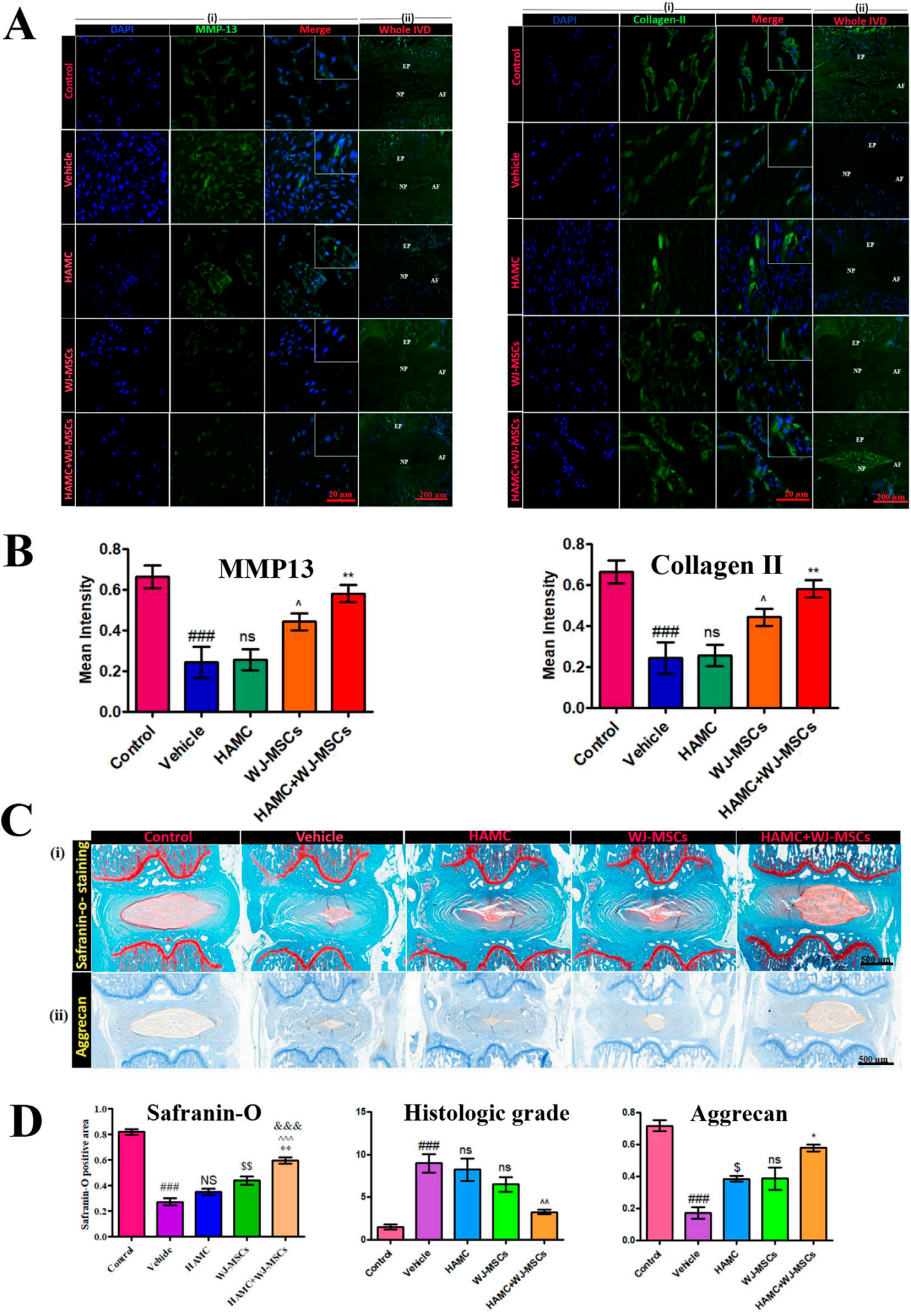
Therapeutic effects of combined hyaluronan -methylcellulose (HAMC) hydrogel with wharton’s jelly-derived mesenchymal stem cells (WJ-MSCs) injection on intervertebral disc degeneration. **(A)** Matrix Metalloproteinase-13 (MMP-13) (left) and Type II collagen (right) immunofluorescence staining results from five experimental groups (Control group: healthy intervertebral disc, Vehicle group: PBS-treated group, hyaluronan–methylcellulose (HAMC) hydrogel group, WJ-MSCs group, HAMC/WJ-MSCs combination therapy group). Nuclei counterstained with DAPI (blue), scale bar: 50 μm. **(B)** Quantitative analysis results. MMP-13 fluorescence intensity: HAMC/WJ-MSCs groups is significantly lower than degenerated group (*p <* 0.01); Type II collagen fluorescence intensity: HAMC/WJ-MSCs group is significantly higher than control group (*p <* 0.01). **(C)** Histological analysis. (i) Safranin-O staining (×200). (ii) Aggrecan immunohistochemistry. **(D)** Histological quantification results. Left: Safranin-O positive area percentage. Middle: histological grading score (0–8 scale, lower scores indicate better outcomes). Right: aggrecan immunoreactivity intensity (relative OD values). Reproduced with permission (Choi et al., 2020). Copyright 2020, The Authors.

Research on synthetic or composite materials containing alginate or HA is also extensive. Choi, U. Y. et al. (Choi et al., 2020) studied the effectiveness of hyaluronan–methylcellulose (HAMC) hydrogels loaded with WJ-MSCs *in vitro* and in a rat coccygeal IVD degeneration model. Combined injection of WJ-MSCs and HAMC promoted IVD regeneration by increasing cell viability, attenuating of the activation of iNOS, MMP-13, ADAMTS4 and COX-2 and significant upregulation of ECM, such as aggrecan and collagen type II (Figure 5). The radiologic and histologic analysis suggest that WJ-MSCs-loaded HAMC promotes IVD repair more effectively than cell injection alone and supports the potential clinical use of HAMC for cell delivery to rescue IDD (Choi et al., 2020) (Figure 5). Jiang et al. (Jiang et al., 2023) utilized a hydrogel composite consisting of sodium alginate (SA), poly (N-isopropylacrylamide) (PNIPAAm), and silicate ceramic (SC) to encapsulate NPCs. This hydrogel demonstrated a sustained release profile of magnesium ions, which was shown to alleviate IDD by promoting the synthesis of ECM and modulating the inflammatory microenvironment associated with IDD.

**FIGURE 6:**
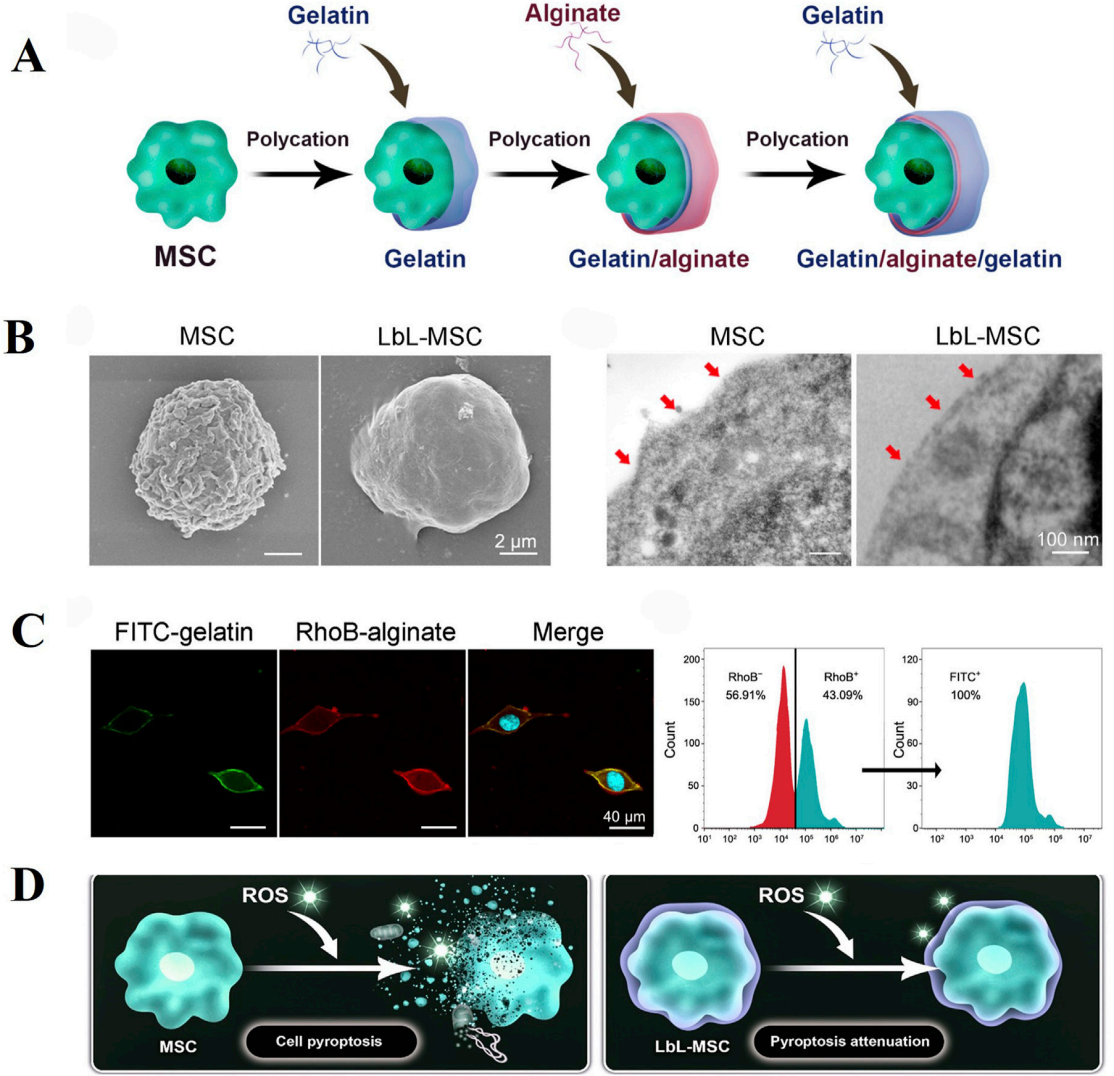
Layer-by-layer (LbL) self-assembly approach can efficiently generate microgel encapsulation of alginate and gelatin over the surface of individual MSC. **(A)** Schematic illustration of LbL encapsulation to achieve single-cell encapsulation by coating alginate and gelatin on cell surface. **(B)** Characterization of LbL encapsulation with Scanning Electron Microscopy (Left) and Transmission Electron Microscopy (Right). **(C)** LbL-MSCs under fluorescent microscope (blue, hoechst; green, FITC-labeled gelatin; red, RhoB-labeled alginate). Cytometry analysis of the ratio of encapsulated cells. RhoB + represented alginate encapsulation, and FITC + represented gelatin encapsulation. **(D)** Microgel coating attenuated pyroptosis activation in MSCs against ROS (Reactive Oxygen Species) stimulation. Reproduced with permission (Huang et al., 2024). Copyright 2024, American Association for the Advancement of Science.

Recent study has shown that encapsulating individual cells is also a promising research approach. Huang et al. (Huang et al., 2024) utilized Layer-by-layer technology to sequentially encapsulate MSCs with gelatin, alginate and gelatin microgels for the treatment of IDD (Figure 6). The microgel coating protects MSCs in the harsh disc microenvironment while preserving crucial cellular functions such as migration, proliferation and differentiation. In rat IDD models, encapsulated MSCs demonstrated prolonged retention within the disc and more effective alleviation of degeneration compared to untreated MSCs alone. However, in the IVD microenvironment, the electrostatic interactions between hydrogel components may be weakened, compromising the microgel’s protective function. Further research is needed on the stability of microgels under acidic and oxidative stress conditions.

**FIGURE 7:**
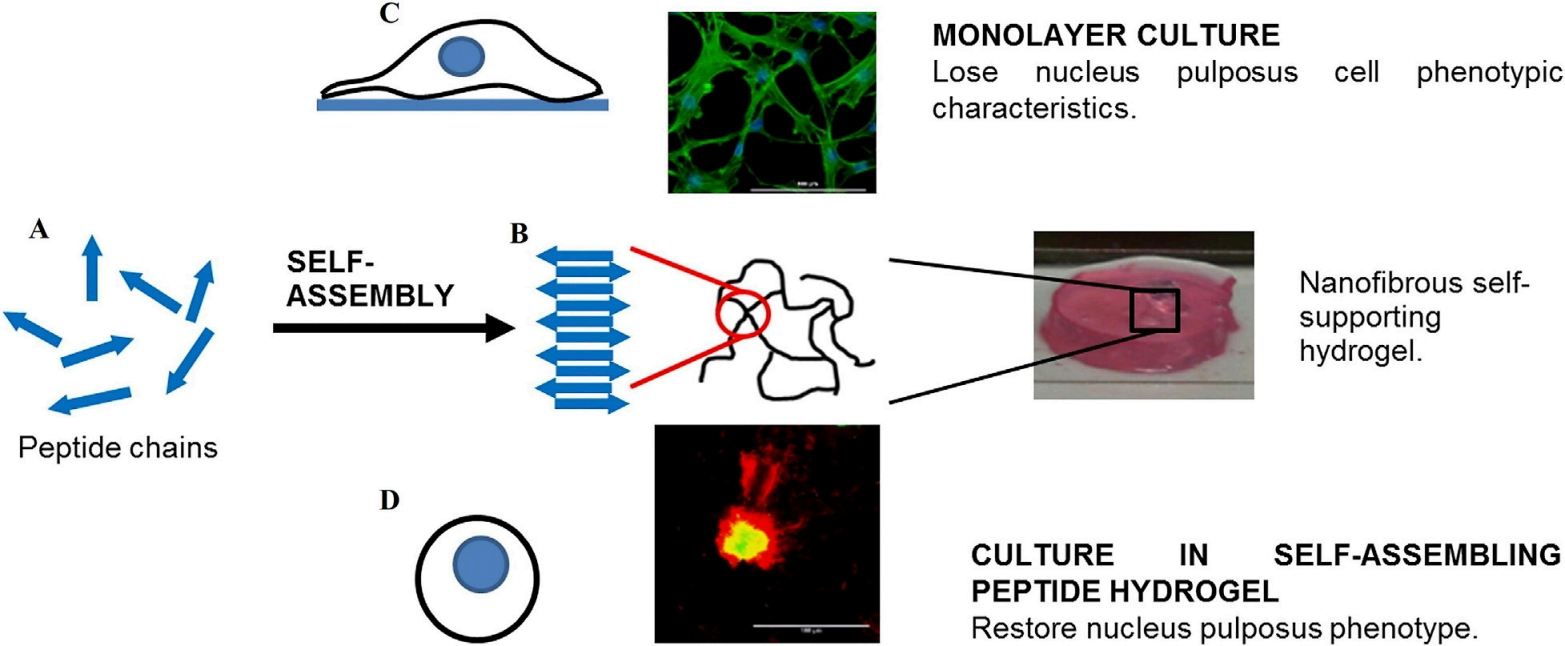
The role of self-assembled peptide hydrogels (SAPH) in maintaining nucleus pulposus cells (NPCs) phenotype. **(A)** Schematic representation of peptide chains undergoing self-assembly. **(B)** Formation of nanofibrous self-supporting hydrogel structure through molecular self-assembly. **(C)** Monolayer culture system where NPCs lose their characteristic phenotype (top panel). **(D)** Three-dimensional culture in SAPH hydrogel that restores and maintains the native NPC phenotype (bottom panel). The comparison demonstrates SAPH’s critical role in providing a biomimetic microenvironment for NPC regeneration. Reproduced with permission (Wan et al., 2016). Copyright 2016, Elsevier.

Self-assembled peptide hydrogels (SAPH) have been extensively explored as cell transporters and scaffolds for NP tissue engineering. SAPH integrates the advantages of both natural and synthetic biomaterials while overcoming their limitations, allowing for the modulation of mechanical and structural properties through peptide sequence modification. After 3D culture of NPCs in the SAPH, Wan et al. (Wan et al., 2016) found that the system could restore the NP phenotype following dedifferentiation through upregulating NP-specific genes (*KRT-8, KRT-18, FOXF-1*) and the SAPH stimulated time-dependent increases in aggrecan and type II collagen deposition (Figure 7). To enhance the mechanical properties of SAPH, Ligorio et al. (Ligorio et al., 2019) investigated the effect of graphene oxide (GO) as nano-filler for the reinforcement of feek (β-sheet forming self-assembling peptide) hydrogels. The results clearly confirm the presence of strong interactions between this peptide and the GO flakes with the peptide coating and forming short thin fibrils on the surface of the flakes. The bulk properties of the final hybrid hydrogels are affected by these strong interactions, which are a function of the peptide content and hydrogel pH.

Synthetic polymers such as polyethylene glycol (PEG), polyvinyl alcohol (PVA), poloxamer, and polyacrylates, with the advantages of precise molecular weight control and reproducible manufacturing, exhibit tailored mechanical properties and appropriate gelation behavior, are widely utilized in the research for improving intervertebral disc degeneration. Kumar et al. (Kumar et al., 2016) created an injectable ultraviolet (UV) photocurable hydrogel containing poly-hydroxyethyl methacrylate (PHEMA), aminopropylmethacrylamide (APMA) and polyacrylic acid (PAA) to culture human BMSCs. They found that their hydrogel promoted the differentiation of hBMSCs into NP cell-like phenotype and the synthesis of proteoglycan and type II collagen under hypoxia and mechanical stimulation. Thorpe et al. (Thorpe et al., 2016) developed injectable thermally triggered hydrogel consisting of n-isopropylacrylamide (NIPAM) and dimethylacrylamide (DMAc). When HMSCs were incorporated into the hydrogel and cultured under 5% O2, the synthesis of proteoglycans and collagen significantly increased. NP markers HIF1α, PAX1 and FOXF1 also significantly increased. Although hydrogels based on PEG match the biomechanical properties of articular cartilage, they are rarely used alone in regenerative medicine because of their poor cell adhesion, which poses a challenge for effective tissue engineering (Jeong et al., 2014).

Tao et al. (Wu et al., 2016) developed an injectable functional hydrogel system manufactured by combining RADA16-I and RADA-KPSS (RADA-KPSS was manufactured by conjugating a bioactive motif derived from BMP-7 (KPSS) onto the C terminal of RADA16-I). Such hydrogel systems can promote the proliferation and differentiation of BMSCs into NPC-like cells and promote the formation of nucleus pulposus cell type II collagen, Sox9 and proteoglycan.

Although the dextran/gelatin hydrogel is mechanically weak as an NP implant, the aldehyde groups in the material could react with the tissue surface, causing the hydrogel to adhere to the tissue surface by a covalent connection, preventing encapsulated MSCs from leaking out (Sakai, 2008). Therefore, the dextran/gelatin hydrogel is still used as vehicles for MSCs (Gan et al., 2016).

Nair et al. (Nair et al., 2015) developed a composite hydrogel for NP tissue engineering, made of CP with CS nanoparticles. This composite hydrogel can promote the viability and adhesion of rat ADMSCs. The viability and chondrogenic differentiation of MSCs was significantly enhanced in presence of CS nanoparticles.

Synthetic and Composite materials, which combine the merits of natural substances and try to address their limitations by enhancing mechanical properties, cellular adhesion, and enabling diverse gelation processes to accommodate different environmental requirements, represent a mainstream direction in current research. However, natural material-based composites face the challenge of balancing crosslinker cytotoxicity with high mechanical strength and long-term stability. Meanwhile, synthetic polymer materials, though tunable in mechanical performance and degradation via molecular design and composite engineering, suffer from poor cell recognition and limited bioactivity (Brinkmann et al., 2023; Kmail et al., 2025). Their long-term safety and functional efficacy require further systematic investigation.

#### 9.2.2 Bio-scaffolds used in IVD repair strategies

In addition to being cell carriers, bio-scaffolds are also widely used in the regeneration and repair of degenerative IVD, such as being an artificial IVD prosthesis to provide support (Ashinsky et al., 2020), loading drugs (Pan et al., 2018) and growth factors (Ligorio et al., 2021) (Table 3). Artificial disc prosthesis for the treatment of degenerative intervertebral disc mainly aims at restoring the mechanical properties of the IVD (such as the height of intervertebral space and local mobility of spine) and also raises requirements for biological properties (such as promoting the growth and differentiation of IVD cells). Depending on the extent of replacement, these living implants are used for complete or partial disc replacement.

**TABLE 3:** Types of bio-scaffold used in IVD repair.

Category		Composition	Load	Ref.
Prosthesis	DAPS	NPCs, AFCs, Agarose, PCL, PEO	-	Ashinsky et al. (2020)
	Biomimetic AF-NP composite	NPCs, AFCs, Alginate, PCL	-	Du et al. (2019)
	IPN scaffolds	Carrageenan, PCL, acetone	-	Chan et al. (2017)
	Viscoelastic-adapted dual-network hydrogel	Phenylboronic acid, GelMA; Polyvinyl alcohol		Cai et al. (2025)
	Multi-laminate AFRPs	Decellularized porcine pericardium, Collagen	-	Borem et al. (2017)
	Alginate scaffold	Alginate, Perfluorotributylamine	-	Sun et al. (2016)
	A photopolymerized composite hydrogel	Poly (ethylene glycol), diethyl triethanolamine, dichloromethane	-	Schmocker et al. (2016)
Carriers	GO- self-assembling peptide hydrogels	GO, self-assembling peptide	TGF-β3	Ligorio et al. (2021)
	ROS-responsive hydrogel	HA, Adipic dihydrazide, PBA, 4-formylphenylboronic acid	TIMP3	Li et al. (2025a)
	miR-5590-SNA@DNAgel	Salmon DNA Salt, SNAs, miR-5590	miR-5590	Qingxin et al. (2023)
	siRNA@G5-PBA@Gel	ODEX, Gelatin,Catechol, PBA, Generation 5 PAMAM, siRNA	siRNA	Chen et al. (2023a)
	ZOGA hydrogels	ZOG,Gelatin,antagomir-204–3p	antagomir-204–3p	Chen et al. (2023b)
	PEAD: heparin delivery system	Heparin, PEAD	GDF-5	Zhu et al. (2019)
	Collagen microcarriers	Collagen	TGF-β1	Steffen et al. (2019)
	Sustained-release biodegradable microspheres	Microspheres	BMP-7	van Dijk et al. (2017)
	Chitosan-hyaluronic acid hydrogel	CS, HA	KGN	Zhu et al. (2017)
	The RADKPS functional peptide	RADA16-I	BMP-7	Wu et al. (2016)
	Chitosan Hydrogel	Chitosan, β-glycerophosphate	Celecoxib	Du et al. (2022)

AFRPs, AF, repair patches; BMP7, Bone Morphogenetic Protein 7. DAPS, Disc-like angle ply structure; GDF-5, Growth differentiation factor 5; GelMA, gelatin methacryloyl; HA, Hyaluronic acid; IPN, interpenetrating network; KGN, kartogenin; PBA, phenylboronic acid; PCL, Poly (ε-caprolactone); PEO, polyethylene oxide; PEAD, Polycation poly (ethylene argininylaspartate diglyceride); RADA16-I, Ac-RADARADARADARADA-NH2; TGF-β3, Transforming growth factor-β3; TGF-β1, Transforming growth factor-β1; SNAs, Spherical Nucleic Acids; ODEX, oxidized dextran; TIMP3, the tissue inhibitor of metalloproteinase-3; ZOG, Zinc-Oxidized Sodium Alginate.

##### 9.2.2.1 Development and design of artificial intervertebral disc prostheses

Multiphasic Tissue-engineered Structures: Ashinsky et al. (Ashinsky et al., 2020) developed a multiphasic tissue-engineered disc-like angle-ply structure (DAPS), using agarose and bovine nucleus pulposus cells (NPCs) to construct the nucleus pulposus (NP) region, and poly (ε-caprolactone) (PCL), polyethylene oxide (PEO), and bovine annulus fibrosus cells (AFCs) to construct the annulus fibrosus (AF) region. This design mimics the multilayer structure of the natural intervertebral disc, providing a more physiologically relevant environment for cell growth. The biomechanical properties of the rat caudal vertebrae differ from those of the human lumbar or cervical vertebrae. The caudal vertebrae primarily bear less physiological load. Therefore, using the rat caudal vertebrae for modeling indeed has certain limitations in biomechanical simulation.

**FIGURE 8:**
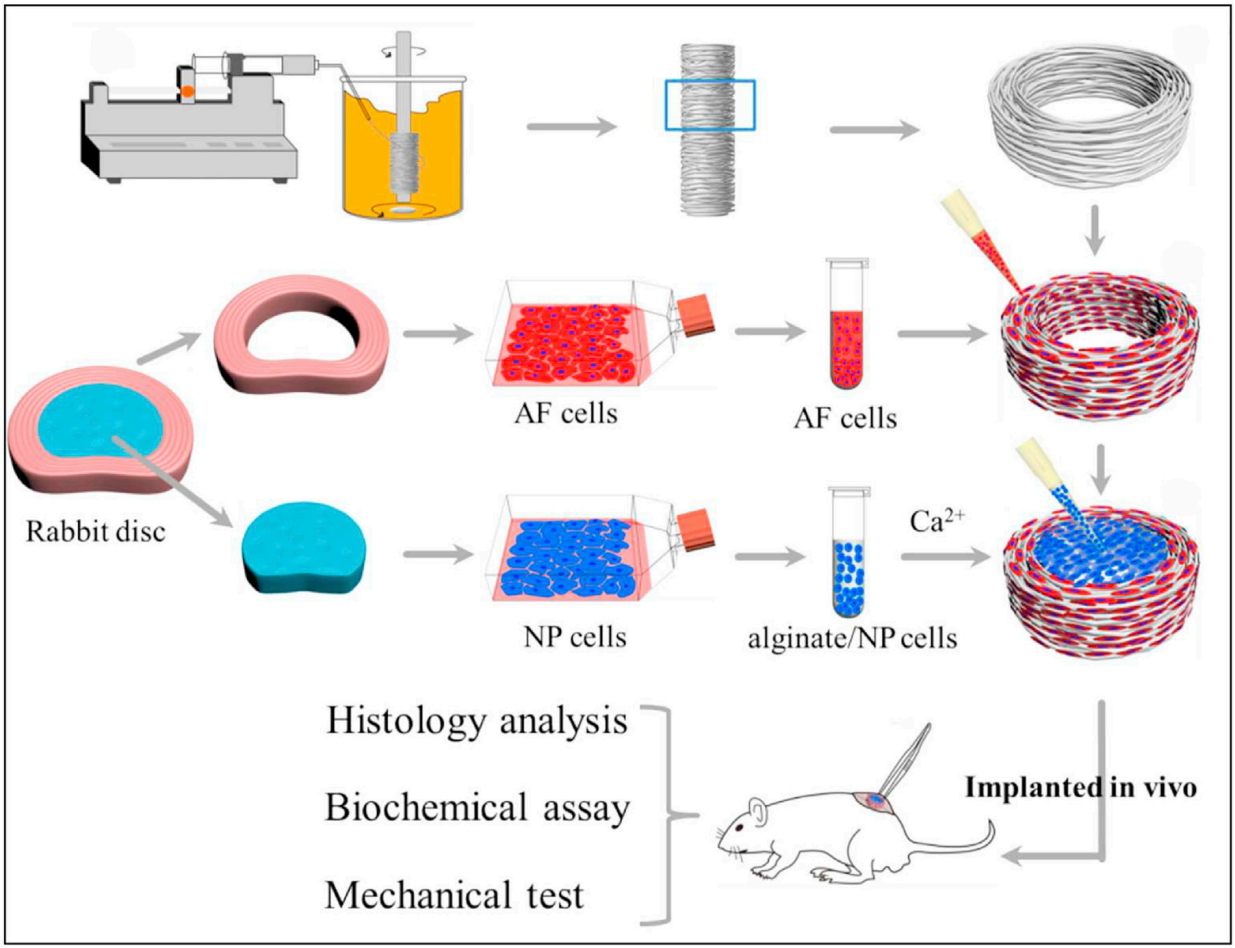
Schematic illustration shows the construction of composite tissue-engineered intervertebral disc (TE-IVD) and the design of *in vitro* study and *in vivo* implantation at nude mice. Reproduced with permission (Du et al., 2019). Copyright 2018, Elsevier.

Biomimetic Composite Materials: Du et al. (Du et al., 2019) designed a biomimetic AF-NP composite with circumferentially oriented poly (ε-caprolactone) microfibers seeded with AF cells, with an alginate hydrogel encapsulating NP cells as the core (Figure 8). This structure not only enhances the mechanical properties of the material but also promotes the oriented growth and functional differentiation of cells. The study did not implant the scaffold into the intervertebral disc. Considering the high-pressure, low-oxygen environment within the intervertebral disc, further exploration is needed to determine whether the cells within the scaffold can proliferate normally under such conditions.

Partial Replacement and Regeneration Strategies: Chan et al. (Chan et al., 2017) successfully increased the stiffness of the material to match that of the native nucleus pulposus by incorporating poly (ε-caprolactone) (PCL) reinforcement into carrageenan gels, providing a possibility for nucleus regeneration. Schmocker et al. (Schmocker et al., 2016) developed a photocurable poly (ethylene glycol) dimethacrylate nanofibrillated cellulose composite hydrogel that can effectively restore intervertebral disc height, offering a new approach for partial replacement. Additionally, Cai et al. Similarly, neither of these studies involved implanting the hydrogel into the load-bearing intervertebral discs of live animals to observe their performance over the long term (Cai et al., 2025) developed a multiscale mechanically adapted viscoelastic double-network hydrogel (PVA-DN) that mimics the mechanical properties of native nucleus pulposus tissue, promoting the proliferation and ECM secretion of NPCs while reducing the inflammatory microenvironment, providing a novel strategy for the repair of intervertebral disc degeneration.

Annulus Fibrosus Repair Patches: Borem et al. (Borem et al., 2017) developed collagen-based multilayer annulus fibrosus repair patches (AFRPs), which demonstrated good mechanical strength and enzymatic stability, while supporting the infiltration and viability of AF cells, providing a new solution for annulus fibrosus repair. Similarly, the laboratory-based mechanical testing conducted in this study cannot reflect the changes in AFRP or suture strength over time, as well as the accumulation of damage, after implantation *in vivo*.

##### 9.2.2.2 Innovations and applications of bioactive hydrogels

Drug and growth factor delivery: Ligorio et al. (Ligorio et al., 2021) developed an injectable graphene oxide (GO) - self-assembling peptide hybrid hydrogel for cell and drug delivery in the NP. Studies have shown that GO flakes can chelate TGF-β3 through strong binding interactions, achieving slow and sustained release. Han et al. (Han et al., 2021) developed GelMA@DMA-MPC hydrogel microspheres that encapsulate diclofenac sodium to achieve sustained drug release while enhancing joint lubrication.

Growth factor delivery vehicles: Zhu et al. (Zhu et al., 2019) developed a growth factor delivery vehicle composed of heparin and synthetic cationic poly (ethylene argininylaspartate diglyceride) (PEAD) for sustained release of GDF5. Steffen et al. (Steffen et al., 2019) cross-linked TGF-β3 to collagen microcarriers using a UV crosslinker, further optimizing the delivery efficiency of growth factors.

**FIGURE 9:**
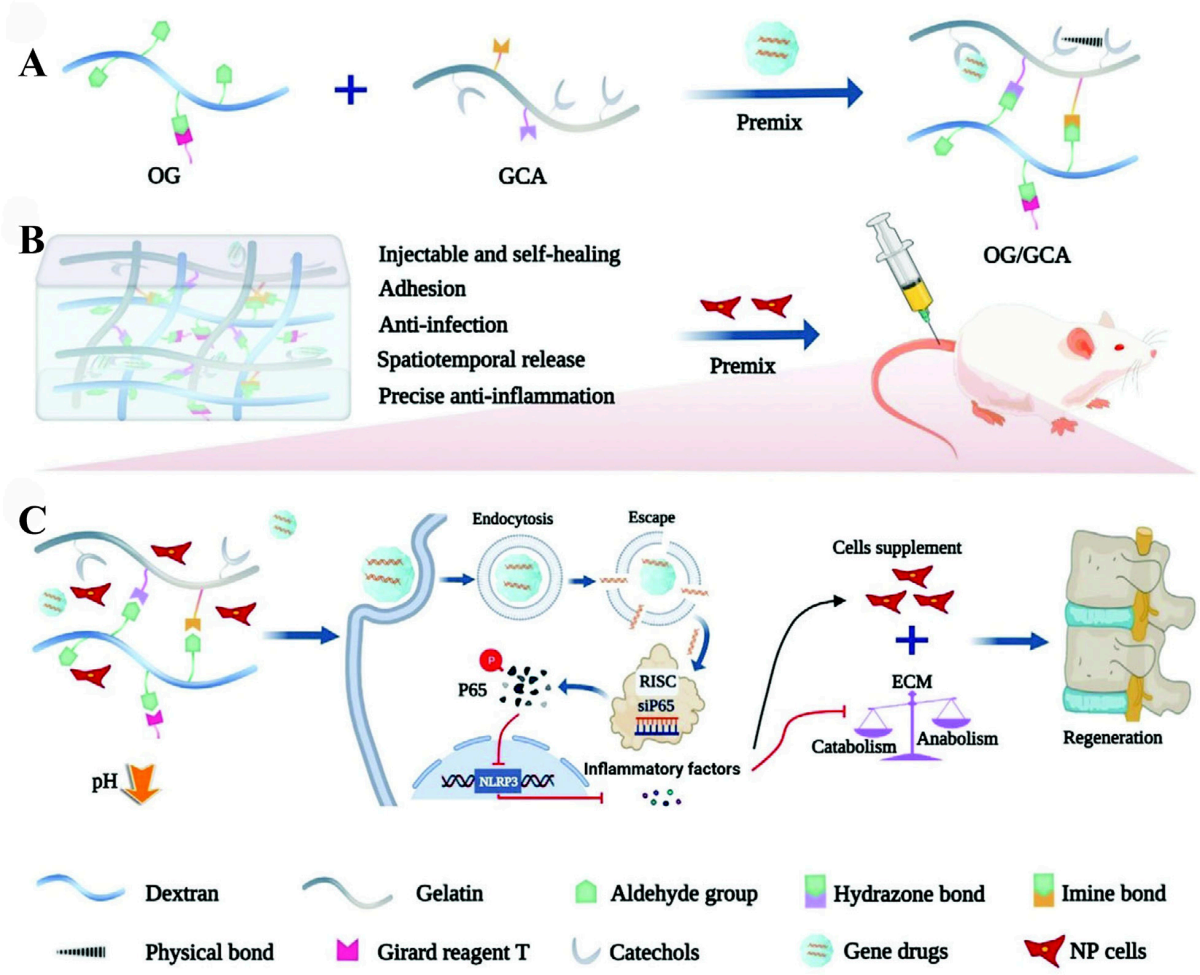
Schematic illustration of the gene-loaded multifunctional hydrogel system for intervertebral disc regeneration. **(A)** shows the injectable self-healing OG/GCA (Oxidized Glycosaminoglycan/Gelatin-Catechol-Adhesive) hydrogel with its four key functions: tissue adhesion, anti-infection, spatiotemporal drug release, and anti-inflammation. **(B)** demonstrates the premixing of hydrogel components (OG and GCA) to form the delivery matrix. **(C)** illustrates the therapeutic mechanism where gene drugs (siP65) are internalized by cells, silence the P65 gene, and subsequently inhibit inflammatory responses. Reproduced with permission (Chen J. et al., 2023). Copyright 2023, the Authors.

Gene therapy and small molecule RNA applications: as research on intervertebral disc degeneration (IDD) deepens, an increasing number of scholars are focusing on gene therapy and the loading of small molecule RNAs. Song et al. (Qingxin et al., 2023) developed a multifunctional DNA hydrogel loaded with miR-5590 to restore the metabolic balance of the extracellular matrix (ECM) by regulating autophagy and apoptosis in NPCs. Chen et al. (Chen J. et al., 2023) designed a high-performance multi-dynamic bond cross-linked hydrogel, siRNA@G5-PBA@Gel, which controls gene expression in a spatiotemporal manner to achieve gene-cell combination therapy for IDD (Figure 9). Tao Chen et al. (Chen T. et al., 2023) developed a high-strength biohydrogel, ZOGA, based on zinc-oxidized sodium alginate-gelatin (ZOG) materials, loaded with antagomir-204–3p (AM) to reduce apoptosis in NPCs by inhibiting miR-204–3p, providing a new strategy for IDD treatment.

In intervertebral disc degeneration (IDD), the imbalance between reactive oxygen species (ROS) generated by oxidative stress and intracellular antioxidant defense mechanisms leads to structural and functional abnormalities, exacerbating disc degeneration (Wang et al., 2023). Several studies have explored the potential of ROS-responsive hydrogels in treating IDD. Li et al. (Li et al., 2025a) developed a benzoboronic acid-functionalized ROS-responsive hydrogel (R-gel) for the controlled release of the tissue inhibitor of metalloproteinase-3 (TIMP3). This hydrogel effectively scavenges ROS and alleviates inflammation-driven disc degeneration. *In vitro* experiments showed that it has low cytotoxicity, reduces ROS levels in nucleus pulposus cells, and mitigates cellular senescence and apoptosis. *In vivo* experiments indicated that it decreases ROS accumulation, inflammatory M1 macrophages, matrix degradation, and neovascularization, significantly improving IDD pathology. Zhang et al. (Zhang et al., 2025) investigated the potential of an allicin-loaded ROS-responsive hydrogel in treating IDD in a rat model. This hydrogel effectively inhibits apoptosis in nucleus pulposus cells by reducing ROS levels and modulating the expression of apoptotic and anti-apoptotic genes, significantly reducing the occurrence of IDD and maintaining disc morphology and matrix integrity.

Bio-scaffolds demonstrate promising potential for intervertebral disc degeneration (IDD) repair, exhibiting favorable biocompatibility, mechanical properties, and regenerative capacity. However, critical challenges hinder clinical translation: (1) current models inadequately replicate the complex disc microenvironment (biomechanical loading, pH gradients, nutrient diffusion); (2) limited clinical data exist, with most studies featuring short-term evaluations (weeks-months) that preclude comprehensive safety and efficacy assessment (Hu et al., 2025). Future research should prioritize development of durable composite materials and establishment of standardized translational protocols to facilitate clinical implementation.

### 9.3 Bioactive factors used in IVD repair strategies

The aim of injecting seed cells into the IVD is to promote the synthesis of proteoglycan and the recovery of disc height. Many active substances, including a variety of cytokines and growth factors, can transform cellular states from catabolic to anabolic and promote cell proliferation and differentiation.

#### 9.3.1 Bioactive factors without carrier

Numerous studies have confirmed the efficacy of bioactive substances in repairing and regenerating degraded intervertebral discs (Table 4). Lykov et al. (Lykov et al., 2020) implanted Wistar rats BMSCs with/without erythropoietin (EPO) into the IDD mouse model. A significant increase in IVD height was found in MSC/EPO group by 21 days of the experiment in comparison with that in MSC group. *In vitro* experiments also proved that EPO could stimulate proliferation under conditions of oxidative stress.

**TABLE 4:** Bioactive factors used in intervertebral disc repair strategies.

Bioactive factors Category		Carriers Category	Research subjects	Ref.
Bioactive factors	EPO		Rat coccygeal vertebrae degeneration model	Lykov et al. (2020)
	MGF		NPCs	Xu et al. (2019b)
	Bleomycin		Rabbit AFSCs	Gao et al. (2018)
	Rapamycin			
	TSG-6		Rat IDD model	Yan et al. (2018)
	Vitamin D		Rat Diabetic model	An et al. (2017)
Bioactive factors with carriers	SOD2, CAT	Gene transfection	Rat coccygeal vertebrae degeneration model	Xiao et al. (2021)
	IL-10, KGN	Gelatin methacryloyl (GelMA) hydrogel	Rat IDD model	Yang et al. (2025b)
	miR-5590	DNAgel	Rat IDD model	Qingxin et al. (2023)
	SLC7A11 modRNA	PVA-tsPBA@SLC7A11 modRNA hydrogels	Rat IDD model	Gao et al. (2024)
	Allicin	Reactive oxygen species -responsive hydrogel	Rat IDD model	Zhang et al. (2025)
	siRNA	G5-PBA@Gel	Rat IDD model	Chen et al. (2023a)
	antagomir-204–3p	ZOGA hydrogels	Rat IDD model	Chen et al. (2023b)
	TGF-β3	Graphene oxide - self-assembling peptide hybrid hydrogels	Bovine NPCs	Ligorio et al. (2021)
	USP15	Gene silencing and overexpression	Degenerative NPCs	Yu et al. (2020)
	GDF5	PEAD:heparin delivery system	Rat coccygeal vertebrae degeneration model	Zhu et al. (2019)
	TGF-β1	Collagen microcarriers; GM@T-NNPs	Dog IDD model; Rat IDD model	Steffen et al. (2019), Zhou et al. (2024)
	Genotropin	Hydromatrix hydrogel, Puramatrix hydrogel	HBMSCs	Hansson et al. (2017)
	OP-1(BMP7)	Sustained-release biodegradable microspheres	Cultured human degenerated NP tissue	van Dijk et al. (2017)
		Gene overexpression	Rabbit IDD model	Xu et al. (2016)
	KGN	Chitosan-hyaluronic acid hydrogel	HADSCS	Zhu et al. (2017)
	IGF-1	Gene silence	Rat IVDs	Li et al. (2013)
	Celecoxib	Chitosan Hydrogel	Rabbit IDD model	Du et al. (2022)
Cocktail therapy	PRP	HA	Bovine IVDs	Russo et al. (2021)
	PRP	-	Rabbit IDD model	Wang et al. (2016)
	iPSC-MSC exosomes	CaCO3/Chitosan Hydrogel	Rat IDD model	Zhan et al. (2025)
	Anti-FAP-scFv-functionalized exosomes	PH-responsive gelatin/oxidized sodium alginate hydrogel	Rat IDD model	Li et al. (2025b)
	Degenerated NPC exosomes		Rat IDD model	Feng et al. (2022)
	CM from hMSCs		Human Disc cells	Hingert et al. (2019)
	CTGF and TGF-β			

CTGF, connective tissue growth factor; Cat, Catalase; CM, conditioned media; EPO, erythropoietin; GM@T-NNPs, Gelatin Methacrylate Microspheres Grafted with TGF-β1-Loaded Neutrophil Membrane-Coated Nanoparticles; IGF-1, Insulin-like growth factors −1; IL-10, Interleukin-10; KGN, kartogenin; MGF, mechano growth factor; PVA-tsPBA@SLC7A11 modRNA, Polyvinyl Alcohol - N1-(4-Boronylbenzyl)-N3-(4-Borophenyl)-N,N,N1,N1,N3,N3 - Hexamethylpropane-1, 3-diamine (tsPBA) Hydrogels Loaded with Solute Carrier Family 7 Member 11 (SLC7A11) Modified Messenger RNA (modRNA); Sod2, Antioxidant’s superoxide dismutase 2; TSG-6, Tumor necrosis factor alpha stimulated gene-6; USP15, Ubiquitin-specific protease 15. iPSC-MSC, Induced Pluripotent Stem Cell-Derived Mesenchymal Stem Cells.

Xu et al. (Xu Q. et al., 2019) found that exogenous mechano growth factor (MGF) partially reversed the effect of mechanical overload on apoptosis of NPCs, and MGF peptide reduced activity of the p38 MAPK pathway of NPCs cultured under mechanical overload.

Apart from cytokines, some drugs have also been studied. Gao et al. (Gao et al., 2018) used cell culture models to study the effects of bleomycin and rapamycin on AFSCs senescence and differentiation. The results showed that the senescence‐associated β‐galactosidase activity the protein expression of P16 AND P21, and inflammatory-related marker gene levels IL-1β, IL-6, and TNF-α were increased in bleomycin-treated AFSCs in a dose-dependent manner. Rapamycin treatment decreased the gene expression of MMP-3, MMP-13, IL-1β, IL-6, TNF-α, and protein levels of P16 and P21 in bleomycin-treated AFSCs suggesting that potential of rapamycin for disc degenerative diseases.

Inflammation is closely related to IDD (Lyu et al., 2021). Several studies have shown that tumor necrosis factor alpha stimulated gene-6 (TSG-6) attenuates inflammatory cascades by inhibiting activation of the TLR2/NF-kappa B signaling pathway (Choi et al., 2011; Liu et al., 2014). Yang et al. (Yan et al., 2018) studied the effects of BMSCs and TSG-6 on IDDs. They found that BMSCs and TSG-6 reduced MMP-3 and MMP-13 expression in IL-1β-treated NPCs, thus restoring the expression of type II collagen and proteoglycan. Furthermore, *in vivo* experiments showed that BMSCs and TSG-6 restored the MRI T2-weighted signal intensity and increased collagen II and aggrecan expression in the degenerated NP tissues. Of note, vitamin D can protect disc degeneration and increase TGF-β and insulin -like growth factor - 1 (IGF-1) (An et al., 2017), which are great benefit to IDD repair.

#### 9.3.2 Bioactive factors with carriers

Although stem cell-based therapy combined with prolonged exposure to growth factor is considered a promising treatment, its effectiveness in alleviating IDD is limited by the short life of the growth factor. To make growth factors work in the long term, some researchers use microcarriers with sustained release (van Dijk et al., 2017) or overexpression of seeded cytokine genes (Xiao et al., 2021).

As research delves deeper into the pathogenesis of intervertebral disc degeneration, the therapeutic potential of exosomes, in part, is increasingly recognized for their capacity to modulate the expression of small non-coding RNAs. The precision of treatment is being enhanced by focusing on these small RNA molecules, which can regulate the expression of proteins associated with the disease. Hydrogels loaded with these RNAs are at the forefront of current research, offering a sustained release mechanism that holds promise for the treatment of intervertebral disc degeneration. Tao Chen et al. (Chen T. et al., 2023) created an engineered high-strength biohydrogel which loaded with antagomir-204–3p (AM). This hydrogel functions to reduce apoptosis in NPCs by inhibiting miR-204–3p, thereby contributing to the amelioration of IDD.

Biodegradable hydrogel is a good choice. Previous studies have shown that TGF-β3 can promote NPCs viability and stimulate aggrecan production (Reza and Nicoll, 2010; Risbud et al., 2006). Ligorio et al. (Ligorio et al., 2021) synthesized a functional 3D scaffold using TGF - β3, graphene oxide and self-assembling peptide. Then, bovine NPCs were cultured on functional scaffolds. By continuous releasing TGF - β3, the results clearly showed that NP-specific gene (*ACAN, COL2A1 and KRT-18*) expression was upregulated while proteoglycan and collagen II were produced and deposited. TGF - β1 also has a protective effect on IVD. For example, TGF - β1 promoted proliferation of NPCs, stimulated synthesis of ECM and inhibited expression of MMP and ADAMTS (Nishida et al., 1999; Wang et al., 2013; Tsuji et al., 2007; Pattison et al., 2001). In addition, the researchers have determined that TGF-Β1 has an anti-inflammatory effect in NPCs (Yang et al., 2015). Steffen et al. (Steffen et al., 2019) found that clinical improvement of the treated dogs IDD after the injection of MSC-TGF-β1-microcarriers. Liang et al. (Zhou et al., 2024) invented a novel hydrogel microsphere system capable of sustaining the release of TGF-β1 for up to 36 days, which has been proven effective in a rat IDD model.

Cell senescence and loss, inflammatory reaction and oxidative stress are closely related to IDD. Therefore, cell replenishment, inhibition of inflammatory response and oxidative stress may contribute to the treatment of IDD (Zhang et al., 2020). Studies have shown that over-expression of either Sod2 or Cat by hADSCs can reduce oxidative stress and enhances treatment of systemic inflammation (Domingues et al., 2019). Xiao et al. (Xiao et al., 2021) implanted hADSCs with/without Ad-null/Ad-Sod 2/Ad-Cat into the IDD mouse model. Compared with the IDD group, IDD mice injected with hADSCs showed increased disc height index, MRI index and mean T2 intensities, as well as the attenuated histologic grading of the annulus fibrosus (AF) and NP accompanied by the upregulation of GAG and COL2. Furthermore, the increased expression of IL-1β, IL-6 and TNF-α was reduced in IDD mice injected with hADSCs. Compared with the hADSC + IDD group, the Ad-Sod2 hADSC/Ad-Cat hADSC + IDD groups got the same results as above (p < 0.05) which confirmed the modification of hADSCs by Sod2 and Cat had therapeutic effect on IDD.

Yu et al. (Yu et al., 2020) induced gene silencing and overexpression of ubiquitin-specific protease 15 (USP15) in degenerative NPCs and found that USP15 was upregulated in degenerative NPCs and that its overexpression accelerated the process of apoptosis. Their results show that USP15 exacerbates NP degradation by deubiquitinating and stabilizing FK506-binding protein 5 (FKBP5).

The GDF5 gene is one of the few genes associated with IDD (Williams et al., 2011; Mu et al., 2013). Defects in this gene lead to collagen and proteoglycan abnormalities (Liang et al., 2010). Walsh et al. (Walsh et al., 2004) found that GDF5 injections significantly increased disc height. Zhu et al. (Zhu et al., 2019) designed a unique growth factor delivery vector for sustained GDF5 release, making GDF five more potent. The results showed that the sustained release of GDF5 promoted the differentiation of hADSC into NP-like phenotypes *in vitro*. After GDF5 delivery platform and hADSCs were injected into the intervertebral space of Co7/Co8 and Co8/Co9, the reduction of disc height, water content and NPs structure were slower than that of other treatment groups. GDF5 helps to maintain the structure and functionality of IVD (Feng et al., 2015).

Bone morphogenetic proteins (BMPs) are a class of growth factors that stimulate the production of bone and cartilage tissue. Bone morphogenetic protein-7 (also known as osteogenesis protein-1, OP-1), a member of the transforming growth factor b (TGF-b) superfamily, shows potential for disc regeneration (Masuda, 2008). OP-1 has stimulating effects on bovine cultured *in vitro* and rabbit NP cells cultured in alginate and can increase the production of agglutinins and type II collagen (Zhang et al., 2004; Masuda et al., 2003). In addition, OP-1 injections have shown potential to increase disc height and enhances the effect of BMSCs on ECM remodeling in animal models of IDD (Xu et al., 2016; Miyamoto et al., 2006; Imai et al., 2007).

IGF-1, a type of hormone similar in molecular structure to insulin, can promote the growth of almost all cells and promote the synthesis of proteoglycan in the NP of adult canine (Thompson et al., 1991). Studies have shown that IGF-l and IGF1R mRNA expression in cultured young intervertebral disc cells is higher than that in mature cells, and the decrease of IGF-l dependent protein polysaccharide synthesis in rat NPCs is due to the downregulation of IGF-IR in late senescence (Osada et al., 1996; Okuda et al., 2001). Genetic variation at the IGFIR gene locus was also associated with IDD (Urano et al., 2008). Li et al. (Li et al., 2013) found that the reduction of IGF1R may lead to accelerated degradation of IVD in mice.

#### 9.3.3 Cocktail therapy

The effect of single cytokine on disc degeneration and regeneration is limited. Multi-factors cocktail therapy may have more therapeutic significance on disc degeneration. Therefore, the study of PRP, conditional medium, exosomes and other cocktail therapy is very hot. PRP is a concentration of growth factors, plasma proteins and platelets isolated from autogenous whole blood by centrifugation. When activated, multiple growth factors, including TGF-β1, IGF-1, platelet-derived growth factor, vascular endothelial growth factor and epidermal growth factor are released from α-granules of platelets, with regenerative effects (Marx, 2001). Release of growth factors can effectively stimulate MSCs to produce type II collagen and agglutinin (Xie et al., 2012).

Wang et al. (Wang et al., 2016) found that the combination of PRP and BMSCs had a better therapeutic effect than PRP alone in the rabbit model of degeneration. Nagae et al. (Nagae et al., 2007) also found that PRP injected within sustained release system exhibited a superior regenerative effect, compared with PRP alone. Therefore, the combination of PRP with sustained release system and MSCs for the treatment of IDD is the future research direction.

Conditioned medium is a medium that has cultured cells, which contains many cytokines secreted by the cells such as growth factors. Hingert et al. (Hingert et al., 2019) compared the effects of CM from hMSCs with the effects of the growth factors CTGF and TGF-β on DCs and hMSCs isolated from patients with low back pain in a 3D *in vitro* model. The findings indicate that CM enhanced the cellular viability and ECM production of DCs while CTGF and the control exhibited nonsignificant differences. The results suggest that CM holds potential to counter the progression of IDD, likely resulting from the combination of all the substances released by the hMSCs.

**FIGURE 10:**
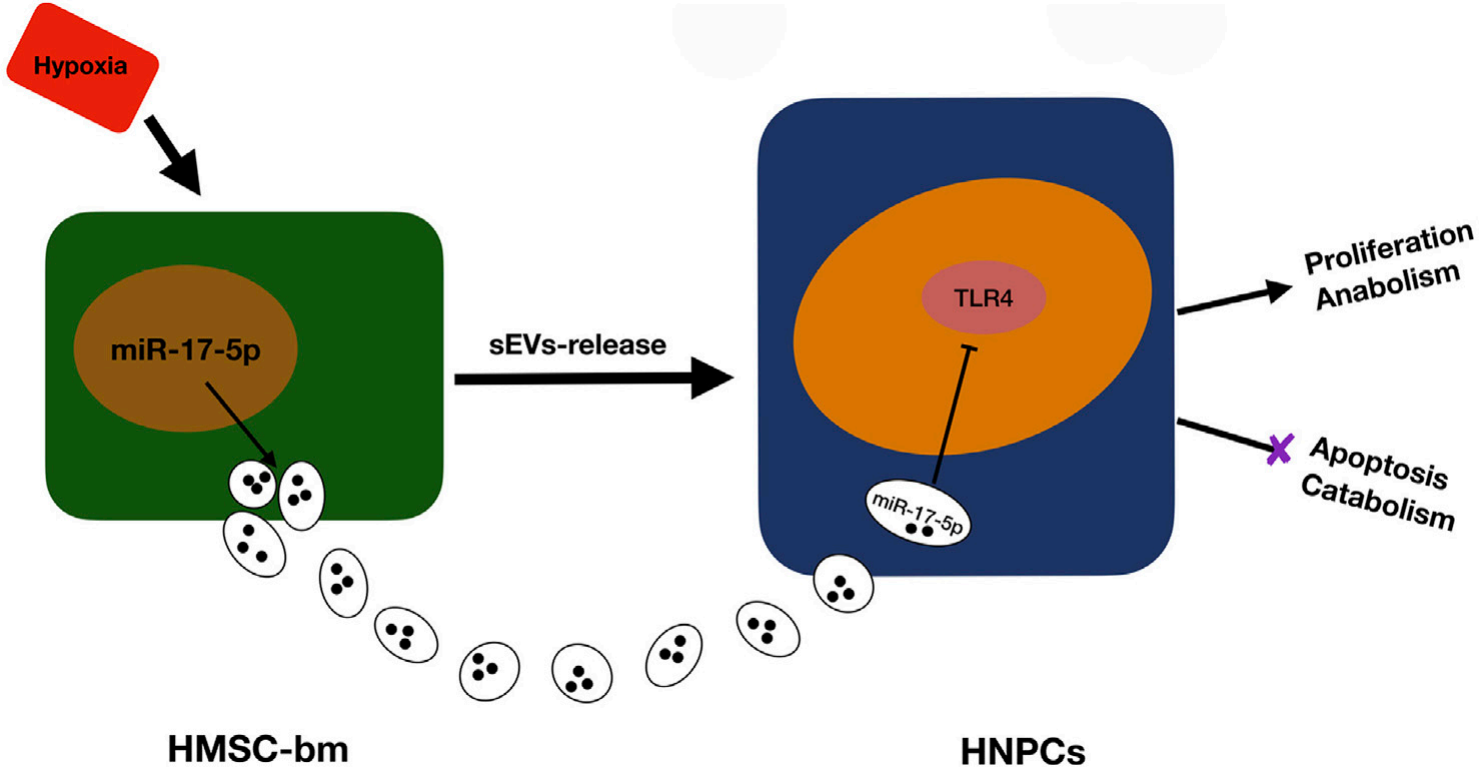
Regulation of NPCs (Nucleus Pulposus Cells) Homeostasis by Hypoxia-derived (Small Extracellular Vesicles) sEVs Enriched with miR-17-5p. Reproduced with permission (Zhou et al., 2022). Copyright 2022, Elsevier.

The exosomes (EXOs, Extracellular vesicles, EVs) are bilayer membranous vesicles, 40–100 nm in diameter, derived from the multivesicular endosomes and released by different cell types into the ECM environment. (Huang et al., 2015; Kourembanas, 2015). Exosomes have been found to include cytokines, proteins, lipids, mRNA, miRNA, non-coding RNA, and ribosomal RNA (Simons and Raposo, 2009). Yu et al. (Yu et al., 2022) revealed that exosomes derived from bone marrow mesenchymal stem cells (BMSCs) promote the survival of nucleus pulposus cells (NPCs) and inhibit ECM degradation via the circ_0050205/miR-665/GPX4 axis, thereby attenuating the progression of intervertebral disc degeneration (IDD). Xia et al. (Xia et al., 2019) found that exosomes play an anti-inflammatory role in pathological NPCs by suppressing inflammatory mediators and NLRP3 inflammasome activation in a rabbit IDD model. Conversely, Feng et al. (Feng et al., 2022) demonstrated that exosomes from degenerated NPCs can exacerbate intervertebral disc degeneration. A major challenge in the clinical application of exosomes is stabilizing their secretion *in vivo*. Xia et al. (Luo et al., 2022) addressed this by using ECM-modified hydrogels (ECM-Gels) to load cartilage endplate stem cells (CESCs) overexpressing sphingosine kinase 2 (Sphk2). After injecting these hydrogels near the cartilaginous endplate (CEP) in rats, they achieved continuous production of CESC-Sphk2-engineered exosomes (CESC-Sphk2-Exos). Zhou et al. (Zhou et al., 2022) isolated hypoxia-derived extracellular vesicles (H-sEVs) from MSCs under hypoxic conditions, which were found to be significantly enriched with miR-17-5p. Both *in vitro* and *in vivo* experiments suggest that miR-17-5p within H-sEVs may modulate the proliferation and matrix synthesis of NPCs via the Toll-like receptor 4 (TLR4) pathway (Figure 10). The CAP-sEXOs@Gel composite hydrogel system (Cartilage Affinity Peptide-modified Exosomes embedded in CaCO_3_/Chitosan Hydrogel), developed by Zhan et al. (Zhan et al., 2025) effectively alleviates inflammation and promotes disc regeneration in intervertebral disc degeneration by targeting the delivery of modified exosomes and neutralizing the acidic environment.

In summary, for direct injection of bioactive factors, their limited *in vivo* persistence may necessitate frequent injections to maintain therapeutic efficacy. To address this issue, hydrogels can be utilized to achieve sustained release. Additionally, the development of condition - responsive hydrogels, such as those responsive to oxidative stress or pH changes, can enable precise release when needed in the intervertebral disc environment, which is a promising direction for future development. Existing therapeutic approaches mostly target single pathological aspects, such as anti - inflammatory or antioxidant effects. However, intervertebral disc degeneration (IDD) is a multifactorial disease that requires a combination of strategies, including anti - inflammatory, antioxidant, and ECM repair actions. As for multifactorial therapies, like platelet - rich plasma (PRP) or exosomes, several challenges exist, including high costs, the risk of immune reactions from allogeneic injections, and difficulties in ensuring the homogeneity of the therapy.

## 10 Conclusion and perspective

LBP is primarily caused by intervertebral disc degeneration (IDD), which has become a hot topic in the field of intervertebral disc tissue engineering. The pathological features of IDD mainly include a reduction in the number of nucleus pulposus cells (NPCs), deterioration of the extracellular matrix (ECM), metabolic imbalance, and structural damage to the intervertebral disc (Vergroesen et al., 2015; Wang et al., 2025; Gu et al., 2025). IDD is also associated with inflammatory mediators such as TNF-α and IL-1, which collectively affect the height and biomechanical properties of the intervertebral disc. Therefore, current therapeutic strategies are mainly focused on these pathological changes.

In terms of therapeutic approaches, the implantation of various types of stem cells not only supplements intervertebral disc cells but also regulates apoptosis and ferroptosis through paracrine or exosome actions, thereby promoting cell growth and enhancing tissue regeneration potential. The transportation of cells or biological components via a carrier can create an environment suitable for load-bearing. For example, hydrogels, with their injectability, biocompatibility, and controlled-release functions, can effectively deliver drugs or stem cells, inhibit oxidative stress and inflammation, and promote ECM regeneration. This carrier can be composed of either natural or synthetic materials. Stem cells from various sources (such as BMSCs and ADSCs) and other bioactive factors (such as TGF-β3 and USP15) have been proven to promote intervertebral disc healing. Recent studies have emphasized the importance of combination therapy, namely, the synergistic action of stem cells and bioactive scaffolds, targeting multifactorial pathological processes (such as oxidative stress, inflammation, ECM degradation, and apoptosis) for comprehensive treatment.

Despite significant progress in the fields of cell therapy and tissue engineering, numerous challenges remain. First, the complexity of the intervertebral disc microenvironment cannot be overlooked. IVD has unique biomechanical and biochemical characteristics (such as pressure, pH, and nutrient transport). Its avascular, hypoxic, and acidic environment limits the survival of drugs and cells, and existing animal models cannot fully simulate the chronic pathological process of human IDD (Chen et al., 2024). Second, the bottleneck in clinical translation is in urgent need of breakthrough. Issues regarding the long-term safety, immunogenicity, and large-scale production of biomaterials have not been properly resolved, and there is a lack of standardized therapeutic efficacy evaluation systems (Gu et al., 2025). In addition, the lack of technical integration is also a major problem at present. Existing therapies mostly target single pathological links, such as anti-inflammatory or antioxidant actions (Mai et al., 2025). However, IDD is a multifactorial disease that requires a combination of anti-inflammatory and ECM repair strategies. Finally, clinical evidence is limited. Most studies are still at the animal experimental stage, and there is a lack of large-scale clinical trials and long-term follow-up data.

Looking to the future, the ideal intervertebral disc repair material should have the following characteristics: good mechanical properties to restore intervertebral space height and bear physiological loads; significant bioactivity to promote the proliferation, differentiation, and signal transduction of intervertebral disc cells; and excellent tissue compatibility to integrate well with surrounding tissues. Future research directions should include: optimizing combination therapies (Mai et al., 2025). by integrating antioxidant, anti-inflammatory, and regenerative materials (such as polysaccharide-nanocomposite materials) and exploring gene editing (such as CRISPR) to enhance stem cell adaptability; accelerating clinical translation by establishing large animal models closer to human IDD (such as pigs and sheep), optimizing targeted delivery systems (such as responsive hydrogels) (Tang et al., 2024), and validating long-term efficacy through multicenter clinical trials (Zhang et al., 2024); and promoting standardization and industrialization by developing unified standards for biomaterial preparation, stem cell sourcing, and therapeutic efficacy evaluation, and fostering industry-university-research cooperation to reduce costs.

From the perspective of clinical application prospects, the application prospects of tissue engineering for the treatment of intervertebral disc degeneration are broad. For patients with early lumbar pain, if conservative treatment can relieve symptoms, there is no need to resort to surgical methods such as injections. The ideal application scenarios include: first, for patients who have not responded to multiple conservative treatments, injecting regenerative materials into the nucleus pulposus to restore intervertebral disc function and alleviate pain; and second, during nucleotomy, injecting relevant regenerative materials to restore or partially restore intervertebral disc function and reduce the probability of reoperation. Although artificial intervertebral disc prostheses are unlikely to achieve the same level of popularity as prostheses in total knee and total hip arthroplasty in the short term, with the advancement of 3D bioprinting technology in the future and the discovery of suitable cell sources and scaffold materials, tissue engineering for the repair and regeneration of degenerative intervertebral discs will become a highly promising option.

## References

[B1] Acosta F. L. Jr. Metz L. Adkisson H. D. IV Liu J. Carruthers-Liebenberg E. Milliman C. (2011). Porcine intervertebral disc repair using allogeneic juvenile articular chondrocytes or mesenchymal stem cells. Tissue Eng. Part A 17 (23-24), 3045–3055. 10.1089/ten.tea.2011.0229 21910592 PMC3226053

[B2] Ahn J. Park E. m. Kim B. J. Kim J. S. Choi B. Lee S. H. (2015). Transplantation of human Wharton's jelly-derived mesenchymal stem cells highly expressing TGFβ receptors in a rabbit model of disc degeneration. Stem Cell Res. Ther. 6, 190. 10.1186/s13287-015-0183-1 26432097 PMC4592544

[B3] An J. L. Zhang W. Zhang J. Lian L. c. Shen Y. Ding W. y. (2017). Vitamin D improves the content of TGF-β and IGF-1 in intervertebral disc of diabetic rats. Exp. Biol. Med. (Maywood) 242 (12), 1254–1261. 10.1177/1535370217707744 28537499 PMC5476342

[B4] Antoniou J. Steffen T. Nelson F. Winterbottom N. Hollander A. P. Poole R. A. (1996). The human lumbar intervertebral disc: evidence for changes in the biosynthesis and denaturation of the extracellular matrix with growth, maturation, ageing, and degeneration. J. Clin. Invest 98 (4), 996–1003. 10.1172/jci118884 8770872 PMC507515

[B5] Arfeen M. Srivastava A. Srivastava N. Khan R. A. Almahmoud S. A. Mohammed H. A. (2024). Design, classification, and adverse effects of NSAIDs: a review on recent advancements. Bioorg Med. Chem. 112, 117899. 10.1016/j.bmc.2024.117899 39217686

[B6] Ashinsky B. G. Gullbrand S. E. Bonnevie E. D. Wang C. Kim D. H. Han L. (2020). Sacrificial fibers improve matrix distribution and micromechanical properties in a tissue-engineered intervertebral disc. Acta Biomater. 111, 232–241. 10.1016/j.actbio.2020.05.019 32447064

[B7] Batsali A. K. Kastrinaki M. C. Papadaki H. A. Pontikoglou C. (2013). Mesenchymal stem cells derived from Wharton's Jelly of the umbilical cord: biological properties and emerging clinical applications. Curr. Stem Cell Res. Ther. 8 (2), 144–155. 10.2174/1574888x11308020005 23279098

[B8] Beeravolu N. Brougham J. Khan I. McKee C. Perez-Cruet M. Chaudhry G. R. (2018). Human umbilical cord derivatives regenerate intervertebral disc. J. Tissue Eng. Regen. Med. 12 (1), e579–e591. 10.1002/term.2330 27690334

[B9] Bertolo A. Häfner S. Taddei A. Baur M. Pötzel T. Steffen F. (2015). Injectable microcarriers as human mesenchymal stem cell support and their application for cartilage and degenerated intervertebral disc repair. Eur. Cell Mater 29, 70–81. discujssion 80-1. 10.22203/ecm.v029a06 25579755

[B10] Bhattarai N. Gunn J. Zhang M. (2010). Chitosan-based hydrogels for controlled, localized drug delivery. Adv. Drug Deliv. Rev. 62 (1), 83–99. 10.1016/j.addr.2009.07.019 19799949

[B11] Borem R. Madeline A. Walters J. Mayo H. Gill S. Mercuri J. (2017). Angle-ply biomaterial scaffold for annulus fibrosus repair replicates native tissue mechanical properties, restores spinal kinematics, and supports cell viability. Acta Biomater. 58, 254–268. 10.1016/j.actbio.2017.06.006 28587986 PMC5832042

[B12] Bowles R. D. Setton L. A. (2017). Biomaterials for intervertebral disc regeneration and repair. Biomaterials 129, 54–67. 10.1016/j.biomaterials.2017.03.013 28324865 PMC5627607

[B13] Brinckmann P. Grootenboer H. (1991). Change of disc height, radial disc bulge, and intradiscal pressure from discectomy. An *in vitro* investigation on human lumbar discs. Spine (Phila Pa 1976) 16 (6), 641–646. 10.1097/00007632-199106000-00008 1862403

[B14] Brinkmann J. Malyaran H. Enezy‐Ulbrich M. A. A. Jung S. Radermacher C. Buhl E. M. (2023). Assessment of fibrin-based hydrogels containing a fibrin-binding peptide to tune mechanical properties and cell responses. Macromol. Mater. Eng. 308 (7), 2200678. 10.1002/mame.202200678

[B15] Bruehlmann S. B. B. Rattner J. R. Matyas J. A. Duncan N. (2002). Regional variations in the cellular matrix of the annulus fibrosus of the intervertebral disc. J. Anat. 201 (2), 159–171. 10.1046/j.1469-7580.2002.00080.x 12220124 PMC1570900

[B16] Buckwalter J. A. (1995). Aging and degeneration of the human intervertebral disc. Spine (Phila Pa 1976) 20 (11), 1307–1314. 10.1097/00007632-199506000-00022 7660243

[B17] Buser Z. Kuelling F. Liu J. Liebenberg E. Thorne K. J. Coughlin D. (2011). Biological and biomechanical effects of fibrin injection into porcine intervertebral discs. Spine (Phila Pa 1976) 36 (18), E1201–E1209. 10.1097/brs.0b013e31820566b2 21325992

[B18] Cai W. Yang F. Yang C. Liu Y. Xu H. Zhang W. (2025). Multiscale mechanical-adapted hydrogels for the repair of intervertebral disc degeneration. Bioact. Mater 48, 336–352. 10.1016/j.bioactmat.2025.02.021 40060142 PMC11889691

[B19] Cassidy J. J. Hiltner A. Baer E. (1989). Hierarchical structure of the intervertebral disc. Connect. Tissue Res. 23 (1), 75–88. 10.3109/03008208909103905 2632144

[B20] Centeno C. Markle J. Dodson E. Stemper I. Williams C. J. Hyzy M. (2017). Treatment of lumbar degenerative disc disease-associated radicular pain with culture-expanded autologous mesenchymal stem cells: a pilot study on safety and efficacy. J. Transl. Med. 15 (1), 197. 10.1186/s12967-017-1300-y 28938891 PMC5610473

[B21] Chan A. H. Boughton P. Ruys A. Oyen M. (2017). An interpenetrating network composite for a regenerative spinal disc application. J. Mech. Behav. Biomed. Mater 65, 842–848. 10.1016/j.jmbbm.2016.10.015 27810730

[B22] Chen J. Zhu H. Xia J. Zhu Y. Xia C. Hu Z. (2023a). High-performance multi-dynamic bond cross-linked hydrogel with spatiotemporal siRNA delivery for gene-cell combination therapy of intervertebral disc degeneration. Adv. Sci. (Weinh) 10 (17), e2206306. 10.1002/advs.202206306 37078785 PMC10265076

[B23] Chen T. Qian Q. Makvandi P. Zare E. N. Chen Q. Chen L. (2023b). Engineered high-strength biohydrogel as a multifunctional platform to deliver nucleic acid for ameliorating intervertebral disc degeneration. Bioact. Mater 25, 107–121. 10.1016/j.bioactmat.2023.01.010 37056255 PMC10088054

[B24] Chen X. Jing S. Xue C. Guan X. (2024). Progress in the application of hydrogels in intervertebral disc repair: a comprehensive review. Curr. Pain Headache Rep. 28 (12), 1333–1348. 10.1007/s11916-024-01296-6 38985414 PMC11666692

[B25] Chen Y. Yang Z. R. Cheng Z. Shi P. Zhang A. Fan J. W. (2025). Injectable hydrogel microspheres promoting inflammation modulation and nucleus pulposus-like differentiation for intervertebral disc regeneration. J. Control Release 380, 599–614. 10.1016/j.jconrel.2025.02.016 39938720

[B26] Choi H. Lee R. H. Bazhanov N. Oh J. Y. Prockop D. J. (2011). Anti-inflammatory protein TSG-6 secreted by activated MSCs attenuates zymosan-induced mouse peritonitis by decreasing TLR2/NF-κB signaling in resident macrophages. Blood 118 (2), 330–338. 10.1182/blood-2010-12-327353 21551236 PMC3138686

[B27] Choi U. Y. Joshi H. P. Payne S. Kim K. T. Kyung J. W. Choi H. (2020). An injectable hyaluronan-methylcellulose (HAMC) hydrogel combined with wharton's jelly-derived mesenchymal stromal cells (WJ-MSCs) promotes degenerative disc repair. Int. J. Mol. Sci. 21 (19), 7391. 10.3390/ijms21197391 33036383 PMC7582266

[B28] Chou A. I. Nicoll S. B. (2009). Characterization of photocrosslinked alginate hydrogels for nucleus pulposus cell encapsulation. J. Biomed. Mater Res. A 91 (1), 187–194. 10.1002/jbm.a.32191 18785646

[B29] Chu G. Shi C. Wang H. Zhang W. Yang H. Li B. (2018). Strategies for annulus fibrosus regeneration: from biological therapies to tissue engineering. Front. Bioeng. Biotechnol. 6, 90. 10.3389/fbioe.2018.00090 30042942 PMC6048238

[B30] Daly C. D. Ghosh P. Zannettino A. C. Badal T. Shimmon R. Jenkin G. (2018). Mesenchymal progenitor cells primed with pentosan polysulfate promote lumbar intervertebral disc regeneration in an ovine model of microdiscectomy. Spine J. 18 (3), 491–506. 10.1016/j.spinee.2017.10.008 29055739

[B31] Day A. J. de la Motte C. A. (2005). Hyaluronan cross-linking: a protective mechanism in inflammation? Trends Immunol. 26 (12), 637–643. 10.1016/j.it.2005.09.009 16214414

[B32] Deng X. Zhao F. Kang B. Zhang X. (2016). Elevated interleukin-6 expression levels are associated with intervertebral disc degeneration. Exp. Ther. Med. 11 (4), 1425–1432. 10.3892/etm.2016.3079 27073460 PMC4812581

[B33] Ding H. Wei J. Zhao Y. Liu Y. Liu L. Cheng L. (2017). Progranulin derived engineered protein Atsttrin suppresses TNF-α-mediated inflammation in intervertebral disc degenerative disease. Oncotarget 8 (65), 109692–109702. 10.18632/oncotarget.22766 29312639 PMC5752552

[B34] Doita M. Kanatani T. Ozaki T. Matsui N. Kurosaka M. Yoshiya S. (2001). Influence of macrophage infiltration of herniated disc tissue on the production of matrix metalloproteinases leading to disc resorption. Spine (Phila Pa 1976) 26 (14), 1522–1527. 10.1097/00007632-200107150-00004 11462080

[B35] Domingues C. C. Kundu N. Kropotova Y. Ahmadi N. Sen S. (2019). Antioxidant-upregulated mesenchymal stem cells reduce inflammation and improve fatty liver disease in diet-induced obesity. Stem Cell Res. Ther. 10 (1), 280. 10.1186/s13287-019-1393-8 31477174 PMC6720095

[B36] Du L. Yang Q. Zhang J. Zhu M. Ma X. Zhang Y. (2019). Engineering a biomimetic integrated scaffold for intervertebral disc replacement. Mater Sci. Eng. C Mater Biol. Appl. 96, 522–529. 10.1016/j.msec.2018.11.087 30606562

[B37] Du Y. Li J. Tang X. Liu Y. Bian G. Shi J. (2022). The thermosensitive injectable celecoxib-loaded chitosan hydrogel for repairing postoperative intervertebral disc defect. Front. Bioeng. Biotechnol. 10, 876157. 10.3389/fbioe.2022.876157 35837544 PMC9274121

[B38] Duance V. C. Crean J. K. G. Sims T. J. Avery N. Smith S. Menage J. (1998). Changes in collagen cross-linking in degenerative disc disease and scoliosis. Spine (Phila Pa 1976) 23 (23), 2545–2551. 10.1097/00007632-199812010-00009 9854753

[B39] Dudli S. Ferguson S. J. Haschtmann D. (2014). Severity and pattern of post-traumatic intervertebral disc degeneration depend on the type of injury. Spine J. 14 (7), 1256–1264. 10.1016/j.spinee.2013.07.488 24583791

[B40] Dudli S. Fields A. J. Samartzis D. Karppinen J. Lotz J. C. (2016). Pathobiology of modic changes. Eur. Spine J. 25 (11), 3723–3734. 10.1007/s00586-016-4459-7 26914098 PMC5477843

[B41] Ejtehadifar M. Shamsasenjan K. Movassaghpour A. Akbarzadehlaleh P. Dehdilani N. Abbasi P. (2015). The effect of hypoxia on mesenchymal stem cell biology. Adv. Pharm. Bull. 5 (2), 141–149. 10.15171/apb.2015.021 26236651 PMC4517092

[B42] Fang B. Song Y. Lin Q. Zhao R. C. (2009). Comparison of human post-embryonic, multipotent stem cells derived from various tissues. Biotechnol. Lett. 31 (7), 929–938. 10.1007/s10529-009-9968-6 19305952

[B43] Feng C. Liu H. Yang Y. Huang B. Zhou Y. (2015). Growth and differentiation factor-5 contributes to the structural and functional maintenance of the intervertebral disc. Cell Physiol. Biochem. 35 (1), 1–16. 10.1159/000369670 25547527

[B44] Feng X. Li Y. Su Q. Tan J. (2022). Degenerative nucleus pulposus cells derived exosomes promoted cartilage endplate cells apoptosis and aggravated intervertebral disc degeneration. Front. Mol. Biosci. 9, 835976. 10.3389/fmolb.2022.835976 35359595 PMC8963919

[B45] Feng Y. Egan B. Wang J. (2016). Genetic factors in intervertebral disc degeneration. Genes Dis. 3 (3), 178–185. 10.1016/j.gendis.2016.04.005 27617275 PMC5016799

[B46] Fernandez C. Marionneaux A. Gill S. Mercuri J. (2016). Biomimetic nucleus pulposus scaffold created from bovine caudal intervertebral disc tissue utilizing an optimal decellularization procedure. J. Biomed. Mater Res. A 104 (12), 3093–3106. 10.1002/jbm.a.35858 27507100 PMC5832047

[B47] Fontana G. See E. Pandit A. (2015). Current trends in biologics delivery to restore intervertebral disc anabolism. Adv. Drug Deliv. Rev. 84, 146–158. 10.1016/j.addr.2014.08.008 25174310

[B48] Gan Y. He J. Zhu J. Xu Z. Wang Z. Yan J. (2021). Spatially defined single-cell transcriptional profiling characterizes diverse chondrocyte subtypes and nucleus pulposus progenitors in human intervertebral discs. Bone Res. 9 (1), 37. 10.1038/s41413-021-00163-z 34400611 PMC8368097

[B49] Gan Y. Li S. Li P. Xu Y. Wang L. Zhao C. (2016). A controlled release codelivery system of MSCs encapsulated in dextran/gelatin hydrogel with TGF-β3-loaded nanoparticles for nucleus pulposus regeneration. Stem Cells Int. 2016, 9042019. 10.1155/2016/9042019 27774108 PMC5059651

[B50] Gao C. Ning B. Sang C. Zhang Y. (2018). Rapamycin prevents the intervertebral disc degeneration via inhibiting differentiation and senescence of annulus fibrosus cells. Aging (Albany NY) 10 (1), 131–143. 10.18632/aging.101364 29348392 PMC5811247

[B51] Gao T. Xu G. Ma T. Lu X. Chen K. Luo H. (2024). ROS-responsive injectable hydrogel loaded with slc7a11-modRNA inhibits ferroptosis and mitigates intervertebral disc degeneration in rats. Adv. Healthc. Mater 13 (27), e2401103. 10.1002/adhm.202401103 38691848

[B52] Gruber H. E. Hoelscher G. Ingram J. Norton H. Hanley E. Jr (2013). Increased IL-17 expression in degenerated human discs and increased production in cultured annulus cells exposed to IL-1ß and TNF-α. Biotech. Histochem 88 (6), 302–310. 10.3109/10520295.2013.783235 23627571

[B53] Grunhagen T. Shirazi-Adl A. Fairbank J. C. Urban J. P. (2011). Intervertebral disk nutrition: a review of factors influencing concentrations of nutrients and metabolites. Orthop. Clin. North Am. 42 (4), 465–477. vii. 10.1016/j.ocl.2011.07.010 21944584

[B54] Gu Z. He Y. Xiang H. Qin Q. Cao X. Jiang K. (2025). Self-healing injectable multifunctional hydrogels for intervertebral disc disease. Mater Today Bio 32, 101655. 10.1016/j.mtbio.2025.101655 PMC11957681 40166378

[B55] Guo W. Douma L. Hu M. H. Eglin D. Alini M. Šećerović A. (2022). Hyaluronic acid-based interpenetrating network hydrogel as a cell carrier for nucleus pulposus repair. Carbohydr. Polym. 277, 118828. 10.1016/j.carbpol.2021.118828 34893245

[B56] Han Y. Yang J. Zhao W. Wang H. Sun Y. Chen Y. (2021). Biomimetic injectable hydrogel microspheres with enhanced lubrication and controllable drug release for the treatment of osteoarthritis. Bioact. Mater 6 (10), 3596–3607. 10.1016/j.bioactmat.2021.03.022 33869900 PMC8022850

[B57] Hansson A. Wenger A. Henriksson H. B. Li S. Johansson B. Brisby H. (2017). The direction of human mesenchymal stem cells into the chondrogenic lineage is influenced by the features of hydrogel carriers. Tissue Cell 49 (1), 35–44. 10.1016/j.tice.2016.12.004 28011039

[B58] Hartvigsen J. Hancock M. J. Kongsted A. Louw Q. Ferreira M. L. Genevay S. (2018). What low back pain is and why we need to pay attention. Lancet 391 (10137), 2356–2367. 10.1016/s0140-6736(18)30480-x 29573870

[B59] Helen W. Merry C. L. Blaker J. J. Gough J. E. (2007). Three-dimensional culture of annulus fibrosus cells within PDLLA/Bioglass composite foam scaffolds: assessment of cell attachment, proliferation and extracellular matrix production. Biomaterials 28 (11), 2010–2020. 10.1016/j.biomaterials.2007.01.011 17250887

[B60] Henriksson H. B. Papadimitriou N. Hingert D. Baranto A. Lindahl A. Brisby H. (2019). The traceability of mesenchymal stromal cells after injection into degenerated discs in patients with low back pain. Stem Cells Dev. 28 (17), 1203–1211. 10.1089/scd.2019.0074 31237488

[B61] HingertD.NawilaijaroenP.AldridgeJ.BarantoA.BrisbyH. (2019). Investigation of the effect of secreted factors from mesenchymal stem cells on disc cells from degenerated discs. Cells Tissues Organs 208 (1-2), 76–88. 10.1159/000506350 32092752

[B62] Hou Y. Shi G. Shi J. Xu G. Guo Y. Xu P. (2016). Study design: *in vitro* and *in vivo* assessment of bone morphogenic protein 2 combined with platelet-rich plasma on treatment of disc degeneration. Int. Orthop. 40 (6), 1143–1155. 10.1007/s00264-015-2840-5 26169838

[B63] Hoy D. Bain C. Williams G. March L. Brooks P. Blyth F. (2012). A systematic review of the global prevalence of low back pain. Arthritis Rheum. 64 (6), 2028–2037. 10.1002/art.34347 22231424

[B64] Hua Z. Zhao Y. Zhang M. Wang Y. Feng H. Wei X. (2025). Research progress on intervertebral disc repair strategies and mechanisms based on hydrogel. J. Biomater. Appl. 39 (10), 1121–1142. 10.1177/08853282251320227 39929142

[B65] Huang B. Li C. Zhou Y. Luo G. Zhang C. (2010). Collagen II/hyaluronan/chondroitin-6-sulfate tri-copolymer scaffold for nucleus pulposus tissue engineering. J. Biomed. Mater Res. B Appl. Biomater. 92 (2), 322–331. 10.1002/jbm.b.31518 19802835

[B66] Huang B. Zhuang Y. Li C. Q. Liu L. T. Zhou Y. (2011). Regeneration of the intervertebral disc with nucleus pulposus cell-seeded collagen II/hyaluronan/chondroitin-6-sulfate tri-copolymer constructs in a rabbit disc degeneration model. Spine (Phila Pa 1976) 36 (26), 2252–2259. 10.1097/brs.0b013e318209fd85 21358466

[B67] Huang G. Shen H. Xu K. Shen Y. Jiale J. Chu G. (2024). Single-cell microgel encapsulation improves the therapeutic efficacy of mesenchymal stem cells in treating intervertebral disc degeneration via inhibiting pyroptosis. Research (Wash D C). 7, 0311. 10.34133/research.0311 38371273 PMC10871001

[B68] Huang L. Ma W. Feng D. Chen H. Cai B. (2015). Exosomes in mesenchymal stem cells, a new therapeutic strategy for cardiovascular diseases? Int. J. Biol. Sci. 11 (2), 238–245. 10.7150/ijbs.10725 25632267 PMC4308409

[B69] Huang X. Cai Y. Chen K. Ren Q. Huang B. Wan G. (2025). Risk factors and treatment strategies for adjacent segment disease following spinal fusion (Review). Mol. Med. Rep. 31 (2), 33. *(Review)* . 10.3892/mmr.2024.13398 39575466 PMC11605282

[B70] Huang Y. C. Urban J. P. Luk K. D. (2014). Intervertebral disc regeneration: do nutrients lead the way? Nat. Rev. Rheumatol. 10 (9), 561–566. 10.1038/nrrheum.2014.91 24914695

[B71] Hussain I. Sloan S. R. Wipplinger C. Navarro-Ramirez R. Zubkov M. Kim E. (2019). Mesenchymal stem cell-seeded high-density collagen gel for annular repair: 6-week results from *in vivo* sheep models. Neurosurgery 85 (2), E350–e359. 10.1093/neuros/nyy523 30476218

[B72] Iatridis J. C. Nicoll S. B. Michalek A. J. Walter B. A. Gupta M. S. (2013). Role of biomechanics in intervertebral disc degeneration and regenerative therapies: what needs repairing in the disc and what are promising biomaterials for its repair? Spine J. 13 (3), 243–262. 10.1016/j.spinee.2012.12.002 23369494 PMC3612376

[B73] Imai Y. Okuma M. An H. S. Nakagawa K. Yamada M. Muehleman C. (2007). Restoration of disc height loss by recombinant human osteogenic protein-1 injection into intervertebral discs undergoing degeneration induced by an intradiscal injection of chondroitinase ABC. Spine (Phila Pa 1976) 32 (11), 1197–1205. 10.1097/brs.0b013e3180574d26 17495776

[B74] Ishiguro H. Kaito T. Yarimitsu S. Hashimoto K. Okada R. Kushioka J. (2019). Intervertebral disc regeneration with an adipose mesenchymal stem cell-derived tissue-engineered construct in a rat nucleotomy model. Acta Biomater. 87, 118–129. 10.1016/j.actbio.2019.01.050 30690206

[B75] Jayasuriya C. T. Chen Q. (2015). Potential benefits and limitations of utilizing chondroprogenitors in cell-based cartilage therapy. Connect. Tissue Res. 56 (4), 265–271. 10.3109/03008207.2015.1040547 26075411 PMC4772698

[B76] Jeong C. G. Francisco A. T. Niu Z. Mancino R. L. Craig S. L. Setton L. A. (2014). Screening of hyaluronic acid-poly(ethylene glycol) composite hydrogels to support intervertebral disc cell biosynthesis using artificial neural network analysis. Acta Biomater. 10 (8), 3421–3430. 10.1016/j.actbio.2014.05.012 24859415 PMC4145863

[B77] Jiang Y. Wang J. Sun D. Liu Z. Qi L. Du M. (2023). A hydrogel reservoir as a self-contained nucleus pulposus cell delivery vehicle for immunoregulation and repair of degenerated intervertebral disc. Acta Biomater. 170, 303–317. 10.1016/j.actbio.2023.08.023 37597680

[B78] Jimbo K. Park J. S. Yokosuka K. Sato K. Nagata K. (2005). Positive feedback loop of interleukin-1β upregulating production of inflammatory mediators in human intervertebral disc cells *in vitro* . J. Neurosurg. Spine 2 (5), 589–595. 10.3171/spi.2005.2.5.0589 15945434

[B79] Karppinen J. Shen F. H. Luk K. D. Andersson G. B. Cheung K. M. Samartzis D. (2011). Management of degenerative disk disease and chronic low back pain. Orthop. Clin. North Am. 42 (4), 513–528. viii. 10.1016/j.ocl.2011.07.009 21944588

[B80] Khan A. N. Jacobsen H. E. Khan J. Filippi C. G. Levine M. Lehman R. A. Jr. (2017). Inflammatory biomarkers of low back pain and disc degeneration: a review. Ann. N. Y. Acad. Sci. 1410 (1), 68–84. 10.1111/nyas.13551 29265416 PMC5744892

[B81] Kirnaz S. Capadona C. Wong T. Goldberg J. L. Medary B. Sommer F. (2022). Fundamentals of intervertebral disc degeneration. World Neurosurg. 157, 264–273. 10.1016/j.wneu.2021.09.066 34929784

[B82] Kmail M. Razak R. Mohd Isa I. L. (2025). Engineering extracellular matrix-based hydrogels for intervertebral disc regeneration. Front. Bioeng. Biotechnol. 13, 1601154. 10.3389/fbioe.2025.1601154 40375978 PMC12078266

[B83] Kondiah P. J. Choonara Y. E. Kondiah P. P. D. Marimuthu T. Kumar P. Du Toit L. (2016). A review of injectable polymeric hydrogel systems for application in bone tissue engineering. Molecules 21 (11), 1580. 10.3390/molecules21111580 27879635 PMC6272998

[B84] Kos N. Gradisnik L. Velnar T. (2019). A brief review of the degenerative intervertebral disc disease. Med. Arch. 73 (6), 421–424. 10.5455/medarh.2019.73.421-424 32082013 PMC7007629

[B85] Kourembanas S. (2015). Exosomes: vehicles of intercellular signaling, biomarkers, and vectors of cell therapy. Annu. Rev. Physiol. 77, 13–27. 10.1146/annurev-physiol-021014-071641 25293529

[B86] Kumar D. Lyness A. Gerges I. Lenardi C. Forsyth N. R. Liu Y. (2016). Stem cell delivery with polymer hydrogel for treatment of intervertebral disc degeneration: from 3D culture to design of the delivery device for minimally invasive therapy. Cell Transpl. 25 (12), 2213–2220. 10.3727/096368916x692618 27452665

[B87] Kumar H. Ha D. H. Lee E. J. Park J. H. Shim J. H. Ahn T. K. (2017). Safety and tolerability of intradiscal implantation of combined autologous adipose-derived mesenchymal stem cells and hyaluronic acid in patients with chronic discogenic low back pain: 1-year follow-up of a phase I study. Stem Cell Res. Ther. 8 (1), 262. 10.1186/s13287-017-0710-3 29141662 PMC5688755

[B88] Le Maitre C. L. Freemont A. J. Hoyland J. A. (2005). The role of interleukin-1 in the pathogenesis of human intervertebral disc degeneration. Arthritis Res. Ther. 7 (4), R732–R745. 10.1186/ar1732 15987475 PMC1175026

[B89] Le Maitre C. L. Pockert A. Buttle D. Freemont A. Hoyland J. (2007). Matrix synthesis and degradation in human intervertebral disc degeneration. Biochem. Soc. Trans. 35 (Pt 4), 652–655. 10.1042/bst0350652 17635113

[B90] Le Visage C. Yang S. H. Kadakia L. Sieber A. N. Kostuik J. P. Leong K. W. (2006). Small intestinal submucosa as a potential bioscaffold for intervertebral disc regeneration. Spine (Phila Pa 1976) 31 (21), 2423–2430. ; discussion 2431. 10.1097/01.brs.0000238684.04792.eb 17023850

[B91] Li B. Zheng X. F. Ni B. B. Yang Y. H. Jiang S. D. Lu H. (2013). Reduced expression of insulin-like growth factor 1 receptor leads to accelerated intervertebral disc degeneration in mice. Int. J. Immunopathol. Pharmacol. 26 (2), 337–347. 10.1177/039463201302600207 23755749

[B92] Li Y. Zhai Y. Wang T. Wang S. Tian Y. Bian Z. (2025b). An anti-FAP-scFv-functionalized exosome-carrying hydrogel delivers SKI mRNA to fibrotic nucleus pulposus cells to alleviate intervertebral disc degeneration by regulating FOXO3. Theranostics 15 (9), 3877–3899. 10.7150/thno.107776 40213661 PMC11980650

[B93] Li Y. Zhang Y. Wang S. Ma X. Dai C. Wang Y. (2025a). Synergistic reversal of inflammation-mediated degeneration in intervertebral discs: phenylboric acid-grafted hyaluronic acid hydrogel as an anti-oxidative vehicle for Timp-3 delivery and promotion of extracellular matrix synthesis. Acta Biomater. 10.1016/j.actbio.2025.06.011 40484295

[B94] Liang C. Li H. Tao Y. Zhou X. Li F. Chen G. (2012). Responses of human adipose-derived mesenchymal stem cells to chemical microenvironment of the intervertebral disc. J. Transl. Med. 10, 49. 10.1186/1479-5876-10-49 22424131 PMC3338074

[B95] Liang H. Ma S. Y. Feng G. Shen F. H. Joshua Li X. (2010). Therapeutic effects of adenovirus-mediated growth and differentiation factor-5 in a mice disc degeneration model induced by annulus needle puncture. Spine J. 10 (1), 32–41. 10.1016/j.spinee.2009.10.006 19926342 PMC2818300

[B96] Ligorio C. O'Brien M. Hodson N. W. Mironov A. Iliut M. Miller A. F. (2021). TGF-β3-loaded graphene oxide - self-assembling peptide hybrid hydrogels as functional 3D scaffolds for the regeneration of the nucleus pulposus. Acta Biomater. 127, 116–130. 10.1016/j.actbio.2021.03.077 33831573

[B97] Ligorio C. Zhou M. Wychowaniec J. K. Zhu X. Bartlam C. Miller A. F. (2019). Graphene oxide containing self-assembling peptide hybrid hydrogels as a potential 3D injectable cell delivery platform for intervertebral disc repair applications. Acta Biomater. 92, 92–103. 10.1016/j.actbio.2019.05.004 31091473 PMC6582688

[B98] Liu Y. Zhang R. Yan K. Chen F. Huang W. Lv B. (2014). Mesenchymal stem cells inhibit lipopolysaccharide-induced inflammatory responses of BV2 microglial cells through TSG-6. J. Neuroinflammation 11, 135. 10.1186/1742-2094-11-135 25088370 PMC4128538

[B99] Liu Y. Zhao Z. Guo C. Huang Z. Zhang W. Ma F. (2023). Application and development of hydrogel biomaterials for the treatment of intervertebral disc degeneration: a literature review. Front. Cell Dev. Biol. 11, 1286223. 10.3389/fcell.2023.1286223 38130952 PMC10733535

[B100] Loibl M. Wuertz‐Kozak K. Vadala G. Lang S. Fairbank J. Urban J. P. (2019). Controversies in regenerative medicine: should intervertebral disc degeneration be treated with mesenchymal stem cells? JOR Spine 2 (1), e1043. 10.1002/jsp2.1043 31463457 PMC6711491

[B101] Luo L. Gong J. Wang Z. Liu Y. Cao J. Qin J. (2022). Injectable cartilage matrix hydrogel loaded with cartilage endplate stem cells engineered to release exosomes for non-invasive treatment of intervertebral disc degeneration. Bioact. Mater 15, 29–43. 10.1016/j.bioactmat.2021.12.007 35386360 PMC8940768

[B102] Lykov A. P. Bondarenko N. A. Poveshchenko O. V. Kim I. I. Surovtseva M. A. Sadykova J. B. (2020). Treatment of intervertebral disc degeneration in wistar rats with mesenchymal stem cells. Bull. Exp. Biol. Med. 168 (4), 578–582. 10.1007/s10517-020-04756-2 32152846

[B103] Lyu F. J. Cui H. Pan H. Mc Cheung K. Cao X. Iatridis J. C. (2021). Painful intervertebral disc degeneration and inflammation: from laboratory evidence to clinical interventions. Bone Res. 9 (1), 7. 10.1038/s41413-020-00125-x 33514693 PMC7846842

[B104] Maher C. Underwood M. Buchbinder R. (2017). Non-specific low back pain. Lancet 389 (10070), 736–747. 10.1016/s0140-6736(16)30970-9 27745712

[B105] Mai Y. Wu S. Zhang P. Chen N. Wu J. Wei F. (2025). The anti-oxidation related bioactive materials for intervertebral disc degeneration regeneration and repair. Bioact. Mater 45, 19–40. 10.1016/j.bioactmat.2024.10.012 39588482 PMC11585838

[B106] Marx R. E. (2001). Platelet-rich plasma (PRP): what is PRP and what is not PRP? Implant Dent. 10 (4), 225–228. 10.1097/00008505-200110000-00002 11813662

[B107] Masuda K. (2008). Biological repair of the degenerated intervertebral disc by the injection of growth factors *.* Eur. Spine J. 17. 441–451. 10.1007/s00586-008-0749-z 19005698 PMC2587664

[B108] Masuda K. Takegami K. An H. Kumano F. Chiba K. Andersson G. B. J. (2003). Recombinant osteogenic protein-1 upregulates extracellular matrix metabolism by rabbit annulus fibrosus and nucleus pulposus cells cultured in alginate beads. J. Orthop. Res. 21 (5), 922–930. 10.1016/s0736-0266(03)00037-8 12919882

[B109] Masuoka K. Michalek A. J. MacLean J. J. Stokes I. A. F. Iatridis J. C. (2007). Different effects of static versus cyclic compressive loading on rat intervertebral disc height and water loss *in vitro* . Spine (Phila Pa 1976) 32 (18), 1974–1979. 10.1097/brs.0b013e318133d591 17700443 PMC2570779

[B110] Meisel H. J. Agarwal N. Hsieh P. C. Skelly A. Park J. B. Brodke D. (2019). Cell therapy for treatment of intervertebral disc degeneration: a systematic review. Glob. Spine J. 9 (1 Suppl. l), 39s–52s. 10.1177/2192568219829024 PMC6512192 31157145

[B111] Mirza S. K. Deyo R. A. (2007). Systematic review of randomized trials comparing lumbar fusion surgery to nonoperative care for treatment of chronic back pain. Spine (Phila Pa 1976) 32 (7), 816–823. 10.1097/01.brs.0000259225.37454.38 17414918

[B112] Miyamoto K. Masuda K. Kim J. G. Inoue N. Akeda K. Andersson G. B. (2006). Intradiscal injections of osteogenic protein-1 restore the viscoelastic properties of degenerated intervertebral discs. Spine J. 6 (6), 692–703. 10.1016/j.spinee.2006.04.014 17088200

[B113] Mizuno H. Roy A. K. Vacanti C. A. Kojima K. Ueda M. Bonassar L. J. (2004). Tissue-engineered composites of anulus fibrosus and nucleus pulposus for intervertebral disc replacement. Spine (Phila Pa 1976) 29 (12), 1290–1297. 10.1097/01.brs.0000128264.46510.27 15187626

[B114] Mochida J. Sakai D. Nakamura Y. Watanabe T. Yamamoto Y. Kato S. (2015). Intervertebral disc repair with activated nucleus pulposus cell transplantation: a three-year, prospective clinical study of its safety. Eur. Cell Mater 29, 202–212. ; discussion 212. 10.22203/ecm.v029a15 25794529

[B115] Mohd Isa I. L. Teoh S. L. Mohd Nor N. H. Mokhtar S. A. (2022). Discogenic low back pain: anatomy, pathophysiology and treatments of intervertebral disc degeneration. Int. J. Mol. Sci. 24 (1), 208. 10.3390/ijms24010208 36613651 PMC9820240

[B116] Molinos M. Almeida C. R. Caldeira J. Cunha C. Gonçalves R. M. Barbosa M. A. (2015). Inflammation in intervertebral disc degeneration and regeneration. J. R. Soc. Interface 12 (108), 20141191. 10.1098/rsif.2014.1191 25673296 PMC4345483

[B117] Mu J. Ge W. Zuo X. Chen Y. Huang C. (2013). Analysis of association between IL-1β, CASP-9, and GDF5 variants and low-back pain in Chinese male soldier: clinical article. J. Neurosurg. Spine 19 (2), 243–247. 10.3171/2013.4.spine12782 23725396

[B118] Nagae M. Ikeda T. Mikami Y. Hase H. Ozawa H. Matsuda K. I. (2007). Intervertebral disc regeneration using platelet-rich plasma and biodegradable gelatin hydrogel microspheres. Tissue Eng. 13 (1), 147–158. 10.1089/ten.2007.13.147 17518588

[B119] Nair M. B. Baranwal G. Vijayan P. Keyan K. S. Jayakumar R. (2015). Composite hydrogel of chitosan-poly(hydroxybutyrate-co-valerate) with chondroitin sulfate nanoparticles for nucleus pulposus tissue engineering. Colloids Surf. B Biointerfaces 136, 84–92. 10.1016/j.colsurfb.2015.08.026 26363270

[B120] Nakielski P. Rybak D. Jezierska-Woźniak K. Rinoldi C. Sinderewicz E. Staszkiewicz-Chodor J. (2023). Minimally invasive intradiscal delivery of BM-MSCs via fibrous microscaffold carriers. ACS Appl. Mater Interfaces 15 (50), 58103–58118. 10.1021/acsami.3c11710 38019273

[B121] Nasto L. A. Ngo K. Leme A. S. Robinson A. R. Dong Q. Roughley P. (2014). Investigating the role of DNA damage in tobacco smoking-induced spine degeneration. Spine J. 14 (3), 416–423. 10.1016/j.spinee.2013.08.034 24211096 PMC3944725

[B122] Nishida K. Kang J. D. Gilbertson L. G. Moon S. H. Suh J. K. Vogt M. T. (1999). Modulation of the biologic activity of the rabbit intervertebral disc by gene therapy: an *in vivo* study of adenovirus-mediated transfer of the human transforming growth factor beta 1 encoding gene. Spine (Phila Pa 1976) 24 (23), 2419–2425. 10.1097/00007632-199912010-00002 10626303

[B123] Noriega D. C. Ardura F. Hernández-Ramajo R. Martín-Ferrero M. Á. Sánchez-Lite I. Toribio B. (2017). Intervertebral disc repair by allogeneic mesenchymal bone marrow cells: a randomized controlled trial. Transplantation 101 (8), 1945–1951. 10.1097/tp.0000000000001484 27661661

[B124] Okuda S. Myoui A. Ariga K. Nakase T. Yonenobu K. Yoshikawa H. (2001). Mechanisms of age-related decline in insulin-like growth factor-I dependent proteoglycan synthesis in rat intervertebral disc cells. Spine (Phila Pa 1976) 26 (22), 2421–2426. 10.1097/00007632-200111150-00005 11707703

[B125] Osada R. Ohshima H. Ishihara H. Yudoh K. Sakai K. Matsui H. (1996). Autocrine/paracrine mechanism of insulin-like growth factor-1 secretion, and the effect of insulin-like growth factor-1 on proteoglycan synthesis in bovine intervertebral discs. J. Orthop. Res. 14 (5), 690–699. 10.1002/jor.1100140503 8893760

[B126] Pan Z. Sun H. Xie B. Xia D. Zhang X. Yu D. (2018). Therapeutic effects of gefitinib-encapsulated thermosensitive injectable hydrogel in intervertebral disc degeneration. Biomaterials 160, 56–68. 10.1016/j.biomaterials.2018.01.016 29396379

[B127] Pattison S. T. Melrose J. Ghosh P. Taylor T. K. F. (2001). REGULATION OF GELATINASE‐A (MMP‐2) PRODUCTION BY OVINE INTERVERTEBRAL DISC NUCLEUS PULPOSUS CELLS GROWN IN ALGINATE BEAD CULTURE BY TRANSFORMING GROWTH FACTOR‐β_1_AND INSULIN LIKE GROWTH FACTOR‐I. Cell Biol. Int. 25 (7), 679–689. 10.1006/cbir.2000.0718 11448107

[B128] Peng C. H. Chen I. H. Yu T. C. Wang J. H. Wu W. T. Yeh K. T. (2024). Risk factors for reoperation after discectomy of lumbar herniated intervertebral disc disease. Tzu Chi Med. J. 36 (3), 298–303. 10.4103/tcmj.tcmj_206_23 38993826 PMC11236073

[B129] Pereira D. R. Silva‐Correia J. Oliveira J. M. Reis R. L. (2013). Hydrogels in acellular and cellular strategies for intervertebral disc regeneration. J. Tissue Eng. Regen. Med. 7 (2), 85–98. 10.1002/term.500 22072398

[B130] Pettine K. A. Murphy M. B. Suzuki R. K. Sand T. T. (2015). Percutaneous injection of autologous bone marrow concentrate cells significantly reduces lumbar discogenic pain through 12 months. Stem Cells 33 (1), 146–156. 10.1002/stem.1845 25187512

[B131] Qingxin S. Kai J. Dandan Z. Linyu J. Xiuyuan C. Yubo F. (2023). Programmable DNA hydrogel provides suitable microenvironment for enhancing autophagy-based therapies in intervertebral disc degeneration treatment. J. Nanobiotechnology 21 (1), 350. 10.1186/s12951-023-02109-5 37759249 PMC10537074

[B132] Reza A. T. Nicoll S. B. (2010). Serum‐free, chemically defined medium with TGF‐β_3_ enhances functional properties of nucleus pulposus cell‐laden carboxymethylcellulose hydrogel constructs. Biotechnol. Bioeng. 105 (2), 384–395. 10.1002/bit.22545 19777586

[B133] Risbud M. V. Di Martino A. Guttapalli A. Seghatoleslami R. Denaro V. Vaccaro A. R. (2006). Toward an optimum system for intervertebral disc organ culture: TGF-beta 3 enhances nucleus pulposus and anulus fibrosus survival and function through modulation of TGF-beta-R expression and ERK signaling. Spine (Phila Pa 1976) 31 (8), 884–890. 10.1097/01.brs.0000209335.57767.b5 16622376

[B134] Risbud M. V. Shapiro I. M. (2014). Role of cytokines in intervertebral disc degeneration: pain and disc content. Nat. Rev. Rheumatol. 10 (1), 44–56. 10.1038/nrrheum.2013.160 24166242 PMC4151534

[B135] Roberts S. Evans H. Trivedi J. Menage J. (2006). Histology and pathology of the human intervertebral disc. J. Bone Jt. Surg. Am. 88 (Suppl. 2), 10–14. 10.2106/jbjs.f.00019 16595436

[B136] Roberts S. Menage J. Urban J. P. (1989). Biochemical and structural properties of the cartilage end-plate and its relation to the intervertebral disc. Spine (Phila Pa 1976) 14 (2), 166–174. 10.1097/00007632-198902000-00005 2922637

[B137] Roughley P. Hoemann C. DesRosiers E. Mwale F. Antoniou J. Alini M. (2006). The potential of chitosan-based gels containing intervertebral disc cells for nucleus pulposus supplementation. Biomaterials 27 (3), 388–396. 10.1016/j.biomaterials.2005.06.037 16125220

[B138] Russo F. Ambrosio L. Ngo K. Vadalà G. Denaro V. Fan Y. (2019). The role of type I diabetes in intervertebral disc degeneration. Spine (Phila Pa 1976) 44 (17), 1177–1185. 10.1097/brs.0000000000003054 30973512 PMC6697217

[B139] Russo F. Ambrosio L. Peroglio M. Guo W. Wangler S. Gewiess J. (2021). A hyaluronan and platelet-rich plasma hydrogel for mesenchymal stem cell delivery in the intervertebral disc: an organ culture study. Int. J. Mol. Sci. 22 (6), 2963. 10.3390/ijms22062963 33803999 PMC7999916

[B140] Russo F. Hartman R. A. Bell K. M. Vo N. Sowa G. A. Kang J. D. (2017). Biomechanical evaluation of transpedicular nucleotomy with intact annulus fibrosus. Spine (Phila Pa 1976) 42 (4), E193–e201. 10.1097/brs.0000000000001762 28207656

[B141] Sakai D. (2008). Future perspectives of cell-based therapy for intervertebral disc disease *.* Eur. Spine J. 17. p. 452–458. 10.1007/s00586-008-0743-5 19005704 PMC2587661

[B142] Sakai D. Mochida J. Iwashina T. Hiyama A. Omi H. Imai M. (2006). Regenerative effects of transplanting mesenchymal stem cells embedded in atelocollagen to the degenerated intervertebral disc. Biomaterials 27 (3), 335–345. 10.1016/j.biomaterials.2005.06.038 16112726

[B143] Sato K. Kikuchi S. Yonezawa T. (1999). *In vivo* intradiscal pressure measurement in healthy individuals and in patients with ongoing back problems. Spine (Phila Pa 1976) 24 (23), 2468–2474. 10.1097/00007632-199912010-00008 10626309

[B144] Schmocker A. Khoushabi A. Frauchiger D. A. Gantenbein B. Schizas C. Moser C. (2016). A photopolymerized composite hydrogel and surgical implanting tool for a nucleus pulposus replacement. Biomaterials 88, 110–119. 10.1016/j.biomaterials.2016.02.015 26976264

[B145] Shi P. Chee A. Liu W. Chou P. H. Zhu J. (2019). Therapeutic effects of cell therapy with neonatal human dermal fibroblasts and rabbit dermal fibroblasts on disc degeneration and inflammation. Spine J. 19 (1), 171–181. 10.1016/j.spinee.2018.08.005 30142460

[B146] Simons M. Raposo G. (2009). Exosomes-vesicular carriers for intercellular communication. Curr. Opin. Cell Biol. 21 (4), 575–581. 10.1016/j.ceb.2009.03.007 19442504

[B147] Smith L. J. Nerurkar N. L. Choi K. S. Harfe B. D. Elliott D. M. (2011). Degeneration and regeneration of the intervertebral disc: lessons from development. Dis. Model Mech. 4 (1), 31–41. 10.1242/dmm.006403 21123625 PMC3008962

[B148] Soltanmohammadi F. Mahmoudi Gharehbaba A. Javadzadeh Y. (2025). Synergistic strategies in tissue engineering: the role of exosomes and decellularized extracellular matrix hydrogels. Biomed. Pharmacother. 188, 118200. 10.1016/j.biopha.2025.118200 40414001

[B149] Stefanakis M. Al-Abbasi M. Harding I. Pollintine P. Dolan P. Tarlton J. (2012). Annulus fissures are mechanically and chemically conducive to the ingrowth of nerves and blood vessels. Spine (Phila Pa 1976) 37 (22), 1883–1891. 10.1097/brs.0b013e318263ba59 22706090

[B150] Steffen F. Bertolo A. Affentranger R. Ferguson S. J. Stoyanov J. (2019). Treatment of naturally degenerated canine lumbosacral intervertebral discs with autologous mesenchymal stromal cells and collagen microcarriers: a prospective clinical study. Cell Transpl. 28 (2), 201–211. 10.1177/0963689718815459 PMC6362527 30488736

[B151] Stirling A. Worthington T. Rafiq M. Lambert P. A. Elliott T. S. (2001). Association between sciatica and Propionibacterium acnes. Lancet 357 (9273), 2024–2025. 10.1016/s0140-6736(00)05109-6 11438138

[B152] Strioga M. Viswanathan S. Darinskas A. Slaby O. Michalek J. (2012). Same or not the same? Comparison of adipose tissue-derived versus bone marrow-derived mesenchymal stem and stromal cells. Stem Cells Dev. 21 (14), 2724–2752. 10.1089/scd.2011.0722 22468918

[B153] Sun Z. Luo B. Liu Z. Huang L. Liu B. Ma T. (2016). Effect of perfluorotributylamine-enriched alginate on nucleus pulposus cell: implications for intervertebral disc regeneration. Biomaterials 82, 34–47. 10.1016/j.biomaterials.2015.12.013 26741882

[B154] Suzuki S. Fujita N. Fujii T. Watanabe K. Yagi M. Tsuji T. (2017). Potential involvement of the IL-6/JAK/STAT3 pathway in the pathogenesis of intervertebral disc degeneration. Spine (Phila Pa 1976) 42 (14), E817–e824. 10.1097/brs.0000000000001982 27879577

[B155] Takatalo J. Karppinen J. Niinimäki J. Taimela S. Näyhä S. Mutanen P. (2011). Does lumbar disc degeneration on magnetic resonance imaging associate with low back symptom severity in young Finnish adults? Spine (Phila Pa 1976) 36 (25), 2180–2189. 10.1097/brs.0b013e3182077122 21358475

[B156] Tang J. Luo Y. Wang Q. Wu J. Wei Y. (2024). Stimuli-responsive delivery systems for intervertebral disc degeneration. Int. J. Nanomedicine 19, 4735–4757. 10.2147/ijn.s463939 38813390 PMC11135562

[B157] Thompson J. P. Oegema T. R. Jr. Bradford D. S. (1991). Stimulation of mature canine intervertebral disc by growth factors. Spine (Phila Pa 1976) 16 (3), 253–260. 10.1097/00007632-199103000-00001 2028297

[B158] Thorpe A. A. Boyes V. Sammon C. Le Maitre C. (2016). Thermally triggered injectable hydrogel, which induces mesenchymal stem cell differentiation to nucleus pulposus cells: potential for regeneration of the intervertebral disc. Acta Biomater. 36, 99–111. 10.1016/j.actbio.2016.03.029 26996377

[B159] Tsuji T. Chiba K. Imabayashi H. Fujita Y. Hosogane N. Okada Y. (2007). Age-related changes in expression of tissue inhibitor of metalloproteinases-3 associated with transition from the notochordal nucleus pulposus to the fibrocartilaginous nucleus pulposus in rabbit intervertebral disc. Spine (Phila Pa 1976) 32 (8), 849–856. 10.1097/01.brs.0000259804.39881.62 17426628

[B160] Twomey L. T. Taylor J. R. (1987). Age changes in lumbar vertebrae and intervertebral discs. Clin. Orthop. Relat. Res. 224 (224), 97–104. 10.1097/00003086-198711000-00013 3665259

[B161] Ukeba D. Sudo H. Tsujimoto T. Ura K. Yamada K. Iwasaki N. (2020). Bone marrow mesenchymal stem cells combined with ultra-purified alginate gel as a regenerative therapeutic strategy after discectomy for degenerated intervertebral discs. EBioMedicine 53, 102698. 10.1016/j.ebiom.2020.102698 32143180 PMC7057222

[B162] Urano T. Narusawa K. Shiraki M. Usui T. Sasaki N. Hosoi T. (2008). Association of a single nucleotide polymorphism in the insulin-like growth factor-1 receptor gene with spinal disc degeneration in postmenopausal Japanese women. Spine (Phila Pa 1976) 33 (11), 1256–1261. 10.1097/brs.0b013e3181715304 18469701

[B163] Urban J. P. McMullin J. F. (1985). Swelling pressure of the inervertebral disc: influence of proteoglycan and collagen contents. Biorheology 22 (2), 145–157. 10.3233/bir-1985-22205 3986322

[B164] Vadala G. Russo F. Ambrosio L. Di Martino A. Papalia R. Denaro V. (2015). BIOTECHNOLOGIES AND BIOMATERIALS IN SPINE SURGERY. J. Biol. Regul. Homeost. Agents 29 (4 Suppl. l), 137–147. 26652500

[B165] Vadalà G. Russo F. Ambrosio L. Loppini M. Denaro V. (2016). Stem cells sources for intervertebral disc regeneration. World J. Stem Cells 8 (5), 185–201. 10.4252/wjsc.v8.i5.185 27247704 PMC4877563

[B166] Vadalà G. Russo F. De Strobel F. Bernardini M. De Benedictis G. M. Cattani C. (2018). Novel stepwise model of intervertebral disc degeneration with intact annulus fibrosus to test regeneration strategies. J. Orthop. Res. 36 (9), 2460–2468. 10.1002/jor.23905 29603340

[B167] Vadalà G. Russo F. Di Martino A. Denaro V. (2015). Intervertebral disc regeneration: from the degenerative cascade to molecular therapy and tissue engineering. J. Tissue Eng. Regen. Med. 9 (6), 679–690. 10.1002/term.1719 23512973

[B168] Vadalà G. Sowa G. Hubert M. Gilbertson L. G. Denaro V. Kang J. D. (2012). Mesenchymal stem cells injection in degenerated intervertebral disc: cell leakage may induce osteophyte formation. J. Tissue Eng. Regen. Med. 6 (5), 348–355. 10.1002/term.433 21671407

[B169] van Dijk B. G. M. Potier E. van Dijk M. Creemers L. B. Ito K. (2017). Osteogenic protein 1 does not stimulate a regenerative effect in cultured human degenerated nucleus pulposus tissue. J. Tissue Eng. Regen. Med. 11 (7), 2127–2135. 10.1002/term.2111 26612824

[B170] Vergroesen P. P. Kingma I. Emanuel K. Hoogendoorn R. Welting T. van Royen B. (2015). Mechanics and biology in intervertebral disc degeneration: a vicious circle. Osteoarthr. Cartil. 23 (7), 1057–1070. 10.1016/j.joca.2015.03.028 25827971

[B171] Vergroesen P. P. van der Veen A. J. van Royen B. J. Kingma I. Smit T. H. (2014). Intradiscal pressure depends on recent loading and correlates with disc height and compressive stiffness. Eur. Spine J. 23 (11), 2359–2368. 10.1007/s00586-014-3450-4 25031105

[B172] Vo N. V. Hartman R. A. Patil P. R. Risbud M. V. Kletsas D. Iatridis J. C. (2016). Molecular mechanisms of biological aging in intervertebral discs. J. Orthop. Res. 34 (8), 1289–1306. 10.1002/jor.23195 26890203 PMC4988945

[B173] Walker B. F. (2000). The prevalence of low back pain: a systematic review of the literature from 1966 to 1998. J. Spinal Disord. 13 (3), 205–217. 10.1097/00002517-200006000-00003 10872758

[B174] Walsh A. J. Bradford D. S. Lotz J. C. (2004). *In vivo* growth factor treatment of degenerated intervertebral discs. Spine (Phila Pa 1976) 29 (2), 156–163. 10.1097/01.brs.0000107231.67854.9f 14722406

[B175] Wan S. Borland S. Richardson S. M. Merry C. L. Saiani A. Gough J. E. (2016). Self-assembling peptide hydrogel for intervertebral disc tissue engineering. Acta Biomater. 46, 29–40. 10.1016/j.actbio.2016.09.033 27677593

[B176] Wang F. Shi R. Cai F. Wang Y. T. Wu X. T. (2015). Stem cell approaches to intervertebral disc regeneration: obstacles from the disc microenvironment. Stem Cells Dev. 24 (21), 2479–2495. 10.1089/scd.2015.0158 26228642

[B177] Wang H. Zhou Y. Huang B. Liu L. T. Liu M. H. Wang J. (2014). Utilization of stem cells in alginate for nucleus pulposus tissue engineering. Tissue Eng. Part A 20 (5-6), 908–920. 10.1089/ten.tea.2012.0703 24102374

[B178] Wang S. L. Yu Y. L. Tang C. L. Lv F. Z. (2013). Effects of TGF-β1 and IL-1β on expression of ADAMTS enzymes and TIMP-3 in human intervertebral disc degeneration. Exp. Ther. Med. 6 (6), 1522–1526. 10.3892/etm.2013.1348 24250727 PMC3829724

[B179] Wang S. Z. Jin J. Y. Guo Y. D. Ma L.-Y. Chang Q. Peng X. G. (2016). Intervertebral disc regeneration using platelet-rich plasma-containing bone marrow-derived mesenchymal stem cells: a preliminary investigation. Mol. Med. Rep. 13 (4), 3475–3481. 10.3892/mmr.2016.4983 26956080 PMC4805096

[B180] Wang X. Huang Y. Yang Y. Tian X. Jin Y. Jiang W. (2025). Polysaccharide-based biomaterials for regenerative therapy in intervertebral disc degeneration. Mater Today Bio 30, 101395. 10.1016/j.mtbio.2024.101395 PMC11699348 39759846

[B181] Wang Y. Cheng H. Wang T. Zhang K. Zhang Y. Kang X. (2023). Oxidative stress in intervertebral disc degeneration: molecular mechanisms, pathogenesis and treatment. Cell Prolif. 56 (9), e13448. 10.1111/cpr.13448 36915968 PMC10472537

[B182] Wei J. N. Cai F. Wang F. Wu X. T. Liu L. Hong X. (2016). Transplantation of CXCR4 overexpressed mesenchymal stem cells augments regeneration in degenerated intervertebral discs. DNA Cell Biol. 35 (5), 241–248. 10.1089/dna.2015.3118 26788981

[B183] Williams F. M. Popham M. Hart D. J. de Schepper E. Bierma-Zeinstra S. Hofman A. (2011). GDF5 single-nucleotide polymorphism rs143383 is associated with lumbar disc degeneration in Northern European women. Arthritis Rheum. 63 (3), 708–712. 10.1002/art.30169 21360499 PMC3498734

[B184] Wismer N. Grad S. Fortunato G. Ferguson S. J. Alini M. Eglin D. (2014). Biodegradable electrospun scaffolds for annulus fibrosus tissue engineering: effect of scaffold structure and composition on annulus fibrosus cells *in vitro* . Tissue Eng. Part A 20 (3-4), 672–682. 10.1089/ten.TEA.2012.0679 24131280

[B185] Wu Y. Jia Z. Liu L. Zhao Y. Li H. Wang C. (2016). Functional self-assembled peptide nanofibers for bone marrow mesenchymal stem cell encapsulation and regeneration in nucleus pulposus. Artif. Organs 40 (6), E112–E119. 10.1111/aor.12694 27153338

[B186] Xia C. Zeng Z. Fang B. Tao M. Gu C. Zheng L. (2019). Mesenchymal stem cell-derived exosomes ameliorate intervertebral disc degeneration via anti-oxidant and anti-inflammatory effects. Free Radic. Biol. Med. 143, 1–15. 10.1016/j.freeradbiomed.2019.07.026 31351174

[B187] Xiao L. Xu S. J. Liu C. Wang J. Hu B. Xu H. G. (2021). Sod2 and catalase improve pathological conditions of intervertebral disc degeneration by modifying human adipose-derived mesenchymal stem cells. Life Sci. 267, 118929. 10.1016/j.lfs.2020.118929 33359244

[B188] Xie X. Wang Y. Zhao C. Guo S. Liu S. Jia W. (2012). Comparative evaluation of MSCs from bone marrow and adipose tissue seeded in PRP-derived scaffold for cartilage regeneration. Biomaterials 33 (29), 7008–7018. 10.1016/j.biomaterials.2012.06.058 22818985

[B189] Xu J. Liu S. Wang S. Qiu P. Chen P. Lin X. (2019a). Decellularised nucleus pulposus as a potential biologic scaffold for disc tissue engineering. Mater Sci. Eng. C Mater Biol. Appl. 99, 1213–1225. 10.1016/j.msec.2019.02.045 30889657

[B190] Xu J. E X. Q. Wang N. X. Wang M. N. Xie H. X. Cao Y. H. (2016). BMP7 enhances the effect of BMSCs on extracellular matrix remodeling in a rabbit model of intervertebral disc degeneration. Febs J. 283 (9), 1689–1700. 10.1111/febs.13695 26929154

[B191] Xu Q. Fang H. Zhao L. Zhang C. Zhang L. Tian B. (2019b). Mechano growth factor attenuates mechanical overload-induced nucleus pulposus cell apoptosis through inhibiting the p38 MAPK pathway. Biosci. Rep. 39 (3). 10.1042/bsr20182462 PMC6438874 30858307

[B192] Yang H. Cao C. Wu C. Yuan C. Gu Q. Shi Q. (2015). TGF-Βl suppresses inflammation in cell therapy for intervertebral disc degeneration. Sci. Rep. 5, 13254. 10.1038/srep13254 26289964 PMC4542522

[B193] Yang H. Tian W. Wang S. Liu X. Wang Z. Hou L. (2018). TSG-6 secreted by bone marrow mesenchymal stem cells attenuates intervertebral disc degeneration by inhibiting the TLR2/NF-κB signaling pathway. Lab. Invest 98 (6), 755–772. 10.1038/s41374-018-0036-5 29483622

[B194] Yang H. Wu J. Liu J. Ebraheim M. Castillo S. Liu X. (2010). Transplanted mesenchymal stem cells with pure fibrinous gelatin-transforming growth factor-β1 decrease rabbit intervertebral disc degeneration. Spine J. 10 (9), 802–810. 10.1016/j.spinee.2010.06.019 20655810

[B195] Yang S. Shi J. Qiao Y. Teng Y. Zhong X. Wu T. (2025b). Harnessing anti-inflammatory and regenerative potential: GelMA hydrogel loaded with IL-10 and kartogenin for intervertebral disc degeneration therapy. ACS Biomater. Sci. Eng. 11 (3), 1486–1497. 10.1021/acsbiomaterials.4c01864 39846724

[B196] Yang Y. Guo J. Cao H. Tian X. Shen H. Niu J. (2025a). Seeds-and-soil inspired hydrogel microspheres: a dual-action antioxidant and cellular therapy for reversing intervertebral disc degeneration. Biomaterials 321, 123326. 10.1016/j.biomaterials.2025.123326 40239592

[B197] Yu B. Shen B. Ba Z. Liu Z. Yuan J. Zhao W. (2020). USP15 promotes the apoptosis of degenerative nucleus pulposus cells by suppressing the PI3K/AKT signalling pathway. J. Cell Mol. Med. 24 (23), 13813–13823. 10.1111/jcmm.15971 33135363 PMC7754067

[B198] Yu C. Li D. Wang C. Xia K. Wang J. Zhou X. (2021). Injectable kartogenin and apocynin loaded micelle enhances the alleviation of intervertebral disc degeneration by adipose-derived stem cell. Bioact. Mater 6 (10), 3568–3579. 10.1016/j.bioactmat.2021.03.018 33842742 PMC8022109

[B199] Yu X. J. Liu Q. K. Lu R. Wang S. X. Xu H. R. Wang Y. G. (2022). Bone marrow mesenchymal stem cell-derived extracellular vesicles carrying circ_0050205 attenuate intervertebral disc degeneration. Oxid. Med. Cell Longev. 2022, 8983667. 10.1155/2022/8983667 35847582 PMC9277161

[B200] Yuan Q. Du L. Xu H. Zhang K. Li Q. Zhang H. (2022). Autologous mesenchymal stromal cells combined with gelatin sponge for repair intervertebral disc defect after discectomy: a preclinical study in a goat model. Front. Biosci. Landmark Ed. 27 (4), 131. 10.31083/j.fbl2704131 35468690

[B201] Zeng C. Yang Q. Zhu M. Du L. Zhang J. Ma X. (2014). Silk fibroin porous scaffolds for nucleus pulposus tissue engineering. Mater Sci. Eng. C Mater Biol. Appl. 37, 232–240. 10.1016/j.msec.2014.01.012 24582244

[B202] Zhan J. Cui Y. Zhang P. Du Y. Hecker P. Zhou S. (2025). Cartilage endplate-targeted engineered exosome releasing and acid neutralizing hydrogel reverses intervertebral disc degeneration. Adv. Healthc. Mater 14 (2), e2403315. 10.1002/adhm.202403315 39555665

[B203] Zhang G. Z. Deng Y. J. Xie Q. Q. Ren E. H. Ma Z. J. He X. G. (2020). Sirtuins and intervertebral disc degeneration: roles in inflammation, oxidative stress, and mitochondrial function. Clin. Chim. Acta 508, 33–42. 10.1016/j.cca.2020.04.016 32348785

[B204] Zhang T. Huang Q. Lu L. Zhou K. Hu K. Gan K. (2025). ROS-Responsive hydrogel loaded with allicin suppresses cell apoptosis for the treatment of intervertebral disc degeneration in a rat model. World Neurosurg. 193, 675–686. 10.1016/j.wneu.2024.10.056 39490768

[B205] Zhang X. Gao X. Yao X. Kang X. (2024). Revolutionizing intervertebral disc regeneration: advances and future directions in three-dimensional bioprinting of hydrogel scaffolds. Int. J. Nanomedicine 19, 10661–10684. 10.2147/ijn.s469302 39464675 PMC11505483

[B206] Zhang Y. An H. S. Song S. Toofanfard M. Masuda K. Andersson G. B. J. (2004). Growth factor osteogenic protein-1: differing effects on cells from three distinct zones in the bovine intervertebral disc. Am. J. Phys. Med. Rehabil. 83 (7), 515–521. 10.1097/01.phm.0000130031.64343.59 15213475

[B207] Zhang Z. Q. Wang C. Yang P. Wang K. (2018). Mesenchymal stem cells induced by microencapsulated chondrocytes on repairing of intervertebral disc degeneration. Orthop. Surg. 10 (4), 328–336. 10.1111/os.12411 30485683 PMC6594535

[B208] Zhou L. Cai F. Zhu H. Xu Y. Tang J. Wang W. (2024). Immune-defensive microspheres promote regeneration of the nucleus pulposus by targeted entrapment of the inflammatory cascade during intervertebral disc degeneration. Bioact. Mater 37, 132–152. 10.1016/j.bioactmat.2024.03.020 38549774 PMC10972768

[B209] Zhou X. Wang J. Fang W. Tao Y. Zhao T. Xia K. (2018). Genipin cross-linked type II collagen/chondroitin sulfate composite hydrogel-like cell delivery system induces differentiation of adipose-derived stem cells and regenerates degenerated nucleus pulposus. Acta Biomater. 71, 496–509. 10.1016/j.actbio.2018.03.019 29555463

[B210] Zhou Z. M. Bao J. P. Peng X. Gao J. W. Vlf C. Zhang C. (2022). Small extracellular vesicles from hypoxic mesenchymal stem cells alleviate intervertebral disc degeneration by delivering miR-17-5p. Acta Biomater. 140, 641–658. 10.1016/j.actbio.2021.11.044 34879291

[B211] Zhu C. Li J. Liu C. Zhou P. Yang H. Li B. (2016). Modulation of the gene expression of annulus fibrosus-derived stem cells using poly(ether carbonate urethane)urea scaffolds of tunable elasticity. Acta Biomater. 29, 228–238. 10.1016/j.actbio.2015.09.039 26432437

[B212] Zhu J. Xia K. Yu W. Wang Y. Hua J. Liu B. (2019). Sustained release of GDF5 from a designed coacervate attenuates disc degeneration in a rat model. Acta Biomater. 86, 300–311. 10.1016/j.actbio.2019.01.028 30660009

[B213] Zhu Y. Tan J. Zhu H. Lin G. Yin F. Wang L. (2017). Development of kartogenin-conjugated chitosan-hyaluronic acid hydrogel for nucleus pulposus regeneration. Biomater. Sci. 5 (4), 784–791. 10.1039/c7bm00001d 28261733

